# Antimicrobial resistance, virulence defects, and evolutionary dynamics of multidrug-resistant *Klebsiella pneumoniae* from human and animal hosts in Central China

**DOI:** 10.1128/spectrum.02269-25

**Published:** 2025-12-17

**Authors:** Wen Sun, Xiaoman Zhu, Xiaomin Pang, Xiangyun Wu, Qian Guo, Linshen Li, Baojing Dou, Yulian Wang

**Affiliations:** 1 National Reference Laboratory of Veterinary Drug Residues and MOA Key Laboratory for Detection of Veterinary Drug Residues, Huazhong Agricultural University 47895, Wuhan, China; 2 Anlu Animal Disease Prevention and Control Center, Anlu, China; 3 MOA Laboratory for Risk Assessment of Quality and Safety of Livestock and Poultry Products, Huazhong Agricultural University 47895, Wuhan, China; Universita degli Studi dell'Insubria, Varese, Italy

**Keywords:** *Klebsiella pneumoniae*, multidrug-resistance, whole-genome sequencing, zoonotic transmission, virulence

## Abstract

The global dissemination of multidrug-resistant *Klebsiella pneumoniae* (MDR-KP) poses a critical One Health challenge. This study investigated the antimicrobial resistance (AMR) profiles, virulence determinants, and zoonotic transmission risks of *K. pneumoniae* isolates from humans (*n* = 10) and animals (*n* = 42), including bovine, porcine, and avian sources (ducks), in Central China. Whole-genome sequencing and phenotypic assays revealed a high prevalence of multidrug resistance (MDR) (92.3%, 48/52) among *K. pneumoniae* isolates, with host-specific resistance rates: 100% in isolates from bovine and ducks, 90.9% in porcine isolates, and 70% in human isolates. Phylogenetic analysis revealed clustering of animal-derived sequence types (ST35, ST101, and ST592) with human clinical isolates, indicating potential cross-species transmission. No convergence of high virulence and MDR was observed. A representative ST11-KL64 strain (KP-HB21H30845) exhibited low virulence despite harboring canonical virulence gene clusters (*iuc*, *rmpA*, and *iroN*). Genetic characterization identified a 45-bp deletion within the *rmpA* promoter region, located within an ISKpn26-formed composite transposon on plasmid pKP845_1, which likely disrupts gene expression. Adjacent to *iroN* genes positioned near ISKpn26 insertion sites may face transcriptional interference, contributing to attenuated virulence phenotypes. The plasmid-borne existence of AMR determinants (*bla*_KPC-2_ and *bla*_CTX-M-65_) and virulence loci (*iuc* and *ybt* on ICEKp) highlights the emerging risk of MDR hypervirulent *K. pneumoniae* (MDR-hvKP). These findings underscore the urgent need for integrated surveillance and antimicrobial stewardship across human-animal interfaces to mitigate MDR-KP dissemination in both veterinary and clinical environments.

**IMPORTANCE:**

MDR *K. pneumoniae* strains (ST35, ST101, and ST592) from animal hosts show genomic linkage to clinical human isolates, signaling interspecies transmission risks. The attenuated virulence of the ST11-KL64 is attributed to ISKpn26-mediated suppression of *rmpA* expression, a key regulator of hypervirulence in *K. pneumoniae*.

## INTRODUCTION

*Klebsiella pneumoniae* (*K. pneumoniae*), an opportunistic pathogen with zoonotic potential—particularly for multidrug-resistant (MDR) and hypervirulent (hv) strains—extensively colonizes the respiratory and gastrointestinal tracts of humans, animals, and their shared environments (1). *K. pneumoniae* has distinct host-adapted pathogenicity: in humans, it primarily causes pneumonia, urinary tract infections, and bacteremia (2, 3), while in animals, it is frequently associated with bovine mastitis, equine pneumonia, and avian respiratory diseases (4, 5). Central China, characterized by livestock production, high population density, and frequent human-animal contact, presents an elevated risk for cross-species transmission of *K. pneumoniae*. Despite this, the molecular mechanisms underlying inter-host transmission dynamics of *K. pneumoniae* in the region remain poorly understood.

Antimicrobial agents, while vital for infection control, have paradoxically driven the evolution and dissemination of multidrug-resistant *K. pneumoniae* (MDR-KP) (6). Designated by the World Health Organization as a priority pathogen requiring urgent global action, MDR-KP presents significant public health challenges due to its broad-spectrum resistance profiles (7). The emergence of extended-spectrum β-lactamase (ESBL)- and carbapenemase-producing strains, with limited effective therapeutic options, highlights the imperative for robust surveillance and targeted intervention (8). This threat is particularly acute in regions such as Central China, where intensive agricultural practices, high antimicrobial usage in livestock, and frequent human-animal interactions elevate the risk of cross-species transmission and resistance spread. Alarmingly, carbapenem-resistance genes (e.g., *bla*_KPC_, *bla*_NDM_, and *bla*_OXA-48_) have formed intricate transmission networks across healthcare and livestock environments through horizontal gene transfer mediated by mobile genetic elements (MGEs) (9, 10).

Molecular typing analyses have revealed the complex evolutionary trajectories of *K. pneumoniae*. Globally, sequence types (STs) such as ST11, ST101, and ST258 are predominant in healthcare-associated infections, with ST101 characterized as a high-risk clonal complex responsible for global nosocomial outbreaks (11). In Asia, ST11 MDR strains dominate clinical settings, and the emergence of ST11-hv-CRKP—defined by hypervirulence and carbapenem resistance—has emerged as a globally concerning superbug lineage (12–14). Recent evidence of identical STs and resistance gene cassettes in animal-derived and human clinical isolates suggests that cross-species transmission plays a pivotal role in antimicrobial resistance evolution (15). Nevertheless, limited clinical data on the genetic relatedness and adaptive evolution of strains in human-livestock ecosystems highlight the need for targeted studies to clarify these dynamics and guide effective control measures.

The pathogenicity of *K. pneumoniae* is intricately linked to the dynamic evolution of its virulence factors. Classical studies have established strong associations between K1/K2 capsular serotypes and hypervirulent phenotypes (16). More recently, regulatory elements such as *rmpA*/*rmpA2* and iron-acquisition systems (e.g., *iuc* and *ybt* gene clusters) have been identified as key contributors to virulence evolution (17). The co-localization of antimicrobial resistance genes and virulence determinants on MGEs, including plasmids and transposons, has facilitated the emergence of multidrug-resistant hypervirulent *K. pneumoniae* (MDR-hvKP), thus further exacerbating the public health challenge (18, 19).

This study employed whole-genome sequencing (WGS) and bioinformatics analyses to characterize *K. pneumoniae* isolates from humans and livestock in Central China, aiming to elucidate the molecular mechanisms of pathogenicity and drug resistance via comparative and phylogenetic analyses while identifying potential transmission networks at the human-animal interface.

## RESULTS

### Genome diversity and lineage associations

MLST analysis of 52 *K*. *pneumoniae* isolates revealed extensive genomic diversity, with 31 different STs identified. ST2410 emerged as the predominant lineage, accounting for 17.3% (9/52) of all isolates. Human-derived isolates displayed a unique ST distribution: the globally disseminated high-risk clone ST11 accounted for 30.0% (3/10) of clinical strains, while animal-associated lineages included ST101 (8.3%, 2/24 in bovine isolates) and ST592 (28.6%, 2/7 in duck isolates), suggesting potential cross-host transmission or shared reservoirs of certain lineages, including ST101 and ST592, which warrant further genomic and epidemiological investigation.

**Fig 1:**
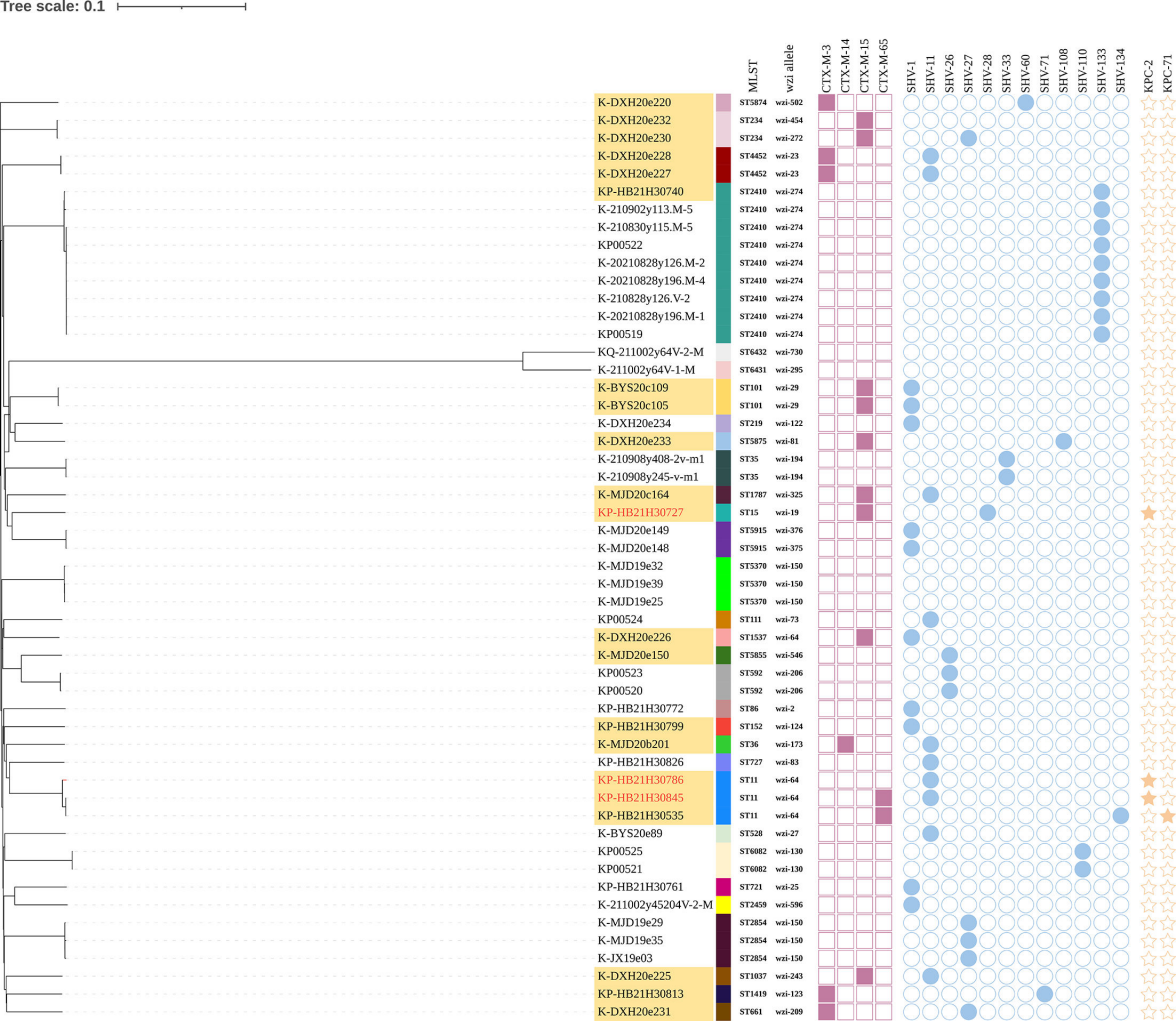
Phylogenetic analysis of 52 *K*. *pneumoniae* isolates with antimicrobial resistance phenotypes and genotypes. ESBL-producing isolates are shaded in orange, carbapenemase producers in red. Colored blocks (purple, blue, and yellow) represent the presence of antibiotic resistance genes, while uncolored blocks indicate gene absence in the corresponding column.

Capsular typing identified 31 distinct *wzi* alleles and 28 KL types. The predominant *wzi* allele was *wzi*-274 (*n* = 9), which was exclusively associated with ST2410 isolates (all harboring KL30 capsular type). This was followed by *wzi*-150 (*n* = 6), linked to ST2854/KL183 and ST5370/KL40. The dominant capsular type was KL30 (21.2%, 11/52), primarily detected in ST2410 and ST234 isolates. Notable minor other notable types included KL64 (*n* = 4, associated with ST11) and KL106 (*n* = 2, associated with ST101) (Fig. 1; Table S1).

### Phylogenetic comparisons of ST11, ST35, ST101, and ST592 *Klebsiella pneumoniae*

**Fig 2:**
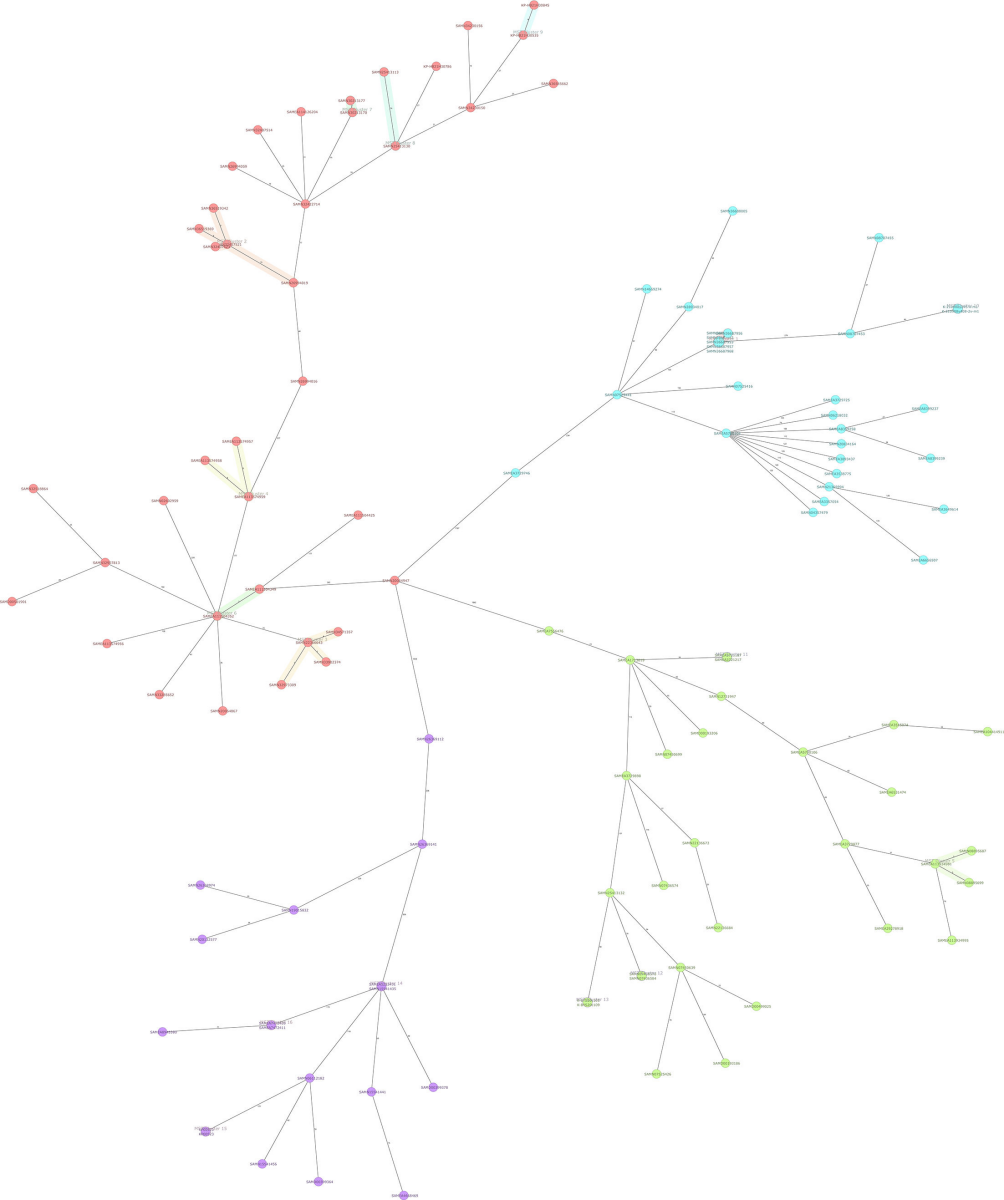
Minimum spanning tree (MST) of *K. pneumoniae* isolates based on core genome MLST (cgMLST, 2,358 loci). Each node represents an isolate, with colors denoting sequence types (STs). The thickness of connecting lines reflects allelic distances between isolates.

Phylogenetic analysis of seven *K. pneumoniae* isolates (ST11, ST35, ST101, ST592) from this study, contextualized against 107 global genomes (94 human, 13 animal) retrieved from the NCBI database (Table S2), resolved 4 major clades stratified by STs. Animal-derived isolates formed tight clusters with human isolate, two ST101 bovine isolates (K-BYS20c105 and K-BYS20c109) clustered closely with human isolates from China (Biosample: SAMN25413132), differing by approximately 90 cgMLST alleles and 500 SNPs, suggesting related but not clonal lineages (outbreak thresholds are typically <25 alleles or <20–50 SNPs). The ST11 and ST592 isolates grouped within human-associated sublineages, further supporting interspecies genetic relatedness (Fig. 2; Fig. S1). These complementary analyses underscore *K. pneumoniae* isolates from animals and humans in central China share close evolutionary relationships with global counterparts, emphasizing the importance of continued One Health genomic surveillance to monitor potential zoonotic dissemination.

### Antimicrobial resistance profiles and genetic determinants

The antimicrobial resistance profiles of 52 *K*. *pneumoniae* isolates exhibited marked host-associated variation. MDR (resistance to ≥3 antimicrobial classes) prevalence reached 92.3% (48/52), with 15.4% (8/52) of isolates resistant to ≥8 antimicrobial classes (Fig. S2). Host-specific resistance patterns emerged among the 52 *K*. *pneumoniae* isolates, reflecting marked variation in antimicrobial profiles. Animal-derived isolates demonstrated high resistance to veterinary antibiotics, including cephalosporins (CEP: 87.5% in bovine isolates and 85.7% in duck isolates; CEF: 58.3% in bovine isolates), aminoglycosides (STR: 83.3% in bovine isolates; KAN: 54.2% in bovine isolates; GEN: 62.5% in bovine isolates), tetracyclines (TET: 54.2% in bovine isolates, 63.6% in porcine isolates, and 71.4% in duck isolates), and quinolones (CIP: >50% in bovine, porcine, and duck isolates). In contrast, human-derived isolates exhibited greater resistance to clinically critical antibiotics, most notably meropenem (40.0%, 4/10) and ciprofloxacin (70.0%, 7/10) (Table 1).

**TABLE 1:** Antibiotic resistance profiles of 52 *K*. *pneumoniae* from different host origins

Antibiotic agents	Antibiotic resistance percentage of *K. pneumoniae* isolates % (number of isolates)				
	Bovine (24)	Porcine (11)	Duck (7)	Human (10)	Total (52)
AMP	100.0 (24)	100.0 (11)	100.0 (7)	100.0 (10)	100.0 (52)
AMC	12.5 (3)	9.1 (1)	28.6 (2)	50.0 (5)	21.2 (11)
CEP	87.5 (21)	27.3 (3)	85.7 (6)	70.0 (7)	71.2 (22)
CEF	58.3 (14)	9.1 (1)	0.0 (0)	60.0 (6)	40.4 (21)
CRO	62.5 (15)	18.2 (2)	14.3 (1)	70.0 (7)	48.1 (23)
MEM	0.0 (0)	0.0 (0)	0.0 (0)	40.0 (4)	7.7 (4)
STR	83.3 (20)	18.2 (2)	14.3 (1)	20.0 (2)	48.1 (23)
KAN	54.2 (13)	36.4 (4)	14.3 (1)	40.0 (4)	42.3 (25)
GEN	62.5 (15)	18.2 (2)	0.0 (0)	40.0 (4)	40.4 (21)
TET	54.2 (13)	63.6 (7)	71.4 (5)	50.0 (5)	57.7 (26)
DOX	50.0 (12)	63.6 (7)	57.1 (4)	50.0 (5)	53.8 (27)
FFC	33.3 (8)	54.5 (6)	42.9 (3)	60.0 (6)	44.2 (28)
CT	0.0 (0)	0.0 (0)	0.0 (0)	0.0 (0)	0.0 (0)
RFX	16.7 (4)	9.1 (1)	42.9 (3)	30.0 (3)	21.2 (11)
CIP	54.2 (13)	54.5 (6)	71.4 (5)	70.0 (7)	59.6 (29)
SOX	75.0 (18)	100.0 (11)	100.0 (7)	90.0 (9)	86.5 (30)
SXT	16.7 (4)	63.6 (7)	100.0 (7)	50.0 (5)	44.2 (28)

ESBL production was detected in 40.4% (21/52) of isolates, with the highest prevalence observed in human (70.0%, 7/10) and bovine (58.3%, 14/24) isolates. Carbapenemase production was restricted to three human isolates (30.0%, 3/10), all of which harbored the *bla*_KPC-2_ gene. Genomic analysis revealed *bla*_SHV_ (88.5%, 46/52) as the dominant β-lactamase gene, followed by *bla*_TEM_ (42.3%, 22/52) and *bla*_CTX-M_ (32.7%, 17/52) (Table S3). Fluoroquinolone resistance (59.6%, 39/52) was significantly associated with plasmid-mediated genes *qnrS1* (51.6%, 16/31; *P* = 0.034) and with the efflux pump component gene *adeF* (45.2%, 14/31; *P* = 0.016). In contrast, other efflux pump genes showed no statistically significant association. Aminoglycoside resistance (75.0%, 39/52) correlated with the presence of enzymatic modification genes *aac(3)-IId* (*P* = 0.013), *aph(3')-Ia* (*P* = 0.04), and *aph(6)-Id* (*P* = 0.017). No colistin resistance (absence of *mcr* gene) was detected, consistent with phenotypic susceptibility (MIC ≤ 2 µg/mL). Resistance gene profiles varied across *K. pneumoniae* lineages. ST101 isolates predominantly harboring *bla*_SHV-1_, while ST11 and ST35 isolates mainly carried *bla*_SHV-11_ and *bla*_SHV-33_, respectively. Isolates harboring *bla*_TEM-1_ were mainly clustered in ST11, ST101, ST4452, ST5370, and ST6082, whereas *bla*_KPC-2_ was almost exclusively found in ST11. Compared to ST35 and ST592 isolates, ST11 and ST101 isolates exhibited a higher burden of resistance genes.

### Virulence phenotypes and genetic determinants

The virulence potential of *K. pneumoniae* isolates was evaluated using phenotypic assays and genomic analysis. Hypermucoviscous (HMV), a key virulence trait, was detected in 9.6% (5/52) of isolates, including two human-derived (20.0%, 2/10), two porcine-derived (18.2%, 2/11), and one duck-derived isolate (14.3%, 1/7). While the fimbrial adhesin gene *mrkD* was universally present (100%, 52/52) and the siderophore biosynthesis gene *entB* was nearly ubiquitous (98.1%, 51/52), canonical hypervirulence markers *magA* (0.0%, 0/52) and *rmpA* (3.9%, 2/52) were largely absent across all isolates (Table 2; Fig. S3A).

**TABLE 2:** Distribution of mucous phenotype-associated virulence genes among 52 *K. pneumoniae* isolates from different origins

Strain origins	Distribution of mucous phenotype-associated virulence genes % (number of isolates)					
	HMV	magA	rmpA	mrkD	fimH-1	entB
Bovine (24)	0.0 (0)	0.0 (0)	0.0 (0)	100.0 (24)	0.0 (0)	100.0 (24)
Human (10)	20.0 (2)	0.0 (0)	20.0 (2)	100.0 (10)	0.0 (0)	90.0 (9)
Porcine (11)	18.2 (2)	0.0 (0)	0.0 (0)	100.0 (11)	0.0 (0)	100.0 (11)
Duck (7)	14.3 (1)	0.0 (0)	0.0 (0)	100.0 (7)	0.0 (0)	100.0 (7)
Total (52)	9.6 (5)	0.0 (0)	3.9 (2)	100.0 (52)	0.0 (0)	98.1 (51)

Mouse lethality assays revealed striking differences in pathogenicity. Among nine representative strains selected based on MLST and HMV status, 88.9% (8/9) displayed low virulence (LD_50_ > 10^5^ CFU) (Table 3; Fig. S3B and S4). Notably, the sole hypervirulent isolate (KP-HB21H30772, human origin, non-ESBL strain) exhibited an HMV phenotype and achieved an LD_50_ of 4.88 × 10^3^ CFU—nearly 1,000-fold higher lethality than the median value of other strains. In contrast, all five ESBL-producing strains showed reduced virulence (LD_50_ values 1–3 log units higher than non-ESBL strains) (Table 3) despite harboring the complete *ybt* (yersiniabactin) and siderophore cluster (*ybt-fyu-irp*). This may reflect a fitness trade-off between antimicrobial resistance and pathogenicity, potentially linked to capsular profile differences, as well as metabolic costs associated with ESBL carriage.

**TABLE 3:** Virulence phenotype of nine strains of *K. pneumoniae*

Strains	Origins	Sequence types	K-locus types	ESBLs	HMV phenotype	LD_50_ (CFU)
K-DXH20e220	Bovine	5874	KL107	Yes	No	1.13 × 10^8^
KP-HB21H30786	Human	11	KL64	Yes	No	1.31 × 10^7^
KP-HB21H30845	Human	11	KL64	Yes	No	5.60 × 10^7^
KP-HB21H30772	Human	86	KL2	No	Yes	4.88 × 10^3^
KP-HB21H30740	Human	2410	KL30	Yes	No	1.07 × 10^7^
K-20210828y196.M-4	Porcine	2410	KL30	No	Yes	7.83 × 10^6^
K-210902y113.M-5	Porcine	2410	KL30	No	No	1.87 × 10^6^
KP00520	Duck	592	KL57	No	No	2.31 × 10^7^
KP00522	Duck	2410	KL30	No	No	9.12 × 10^6^

### Analysis of mobile genetic elements

A total of 27 distinct plasmid replicon types were identified among 52 *K*. *pneumoniae* isolates, spanning incompatibility groups including IncC, IncF, IncHI1, IncL, IncN, IncQ, IncR, IncX, and Col replicons (Col440I, Col440II, ColpVC, and ColRNAI) (Table S4). Predominant replicons included FIB(K) (*n* = 32), FIIK (*n* = 22), HI1B(pNDM-MAR) (*n* = 15), R (*n* = 10), repB (*n* = 10), and FIB(K) (pCAV1099-114) (*n* = 8). Replicon sequence typing (RST) indicated that isolates sharing the same serotype or ST exhibited identical replicon profiles, such as the K5:A-:B- profile in ST2410 and the K12:A-:B- profile in ST101 isolates.

Resistance genes were closely associated with mobile genetic elements: *tet*(A) co-occurred with IncHI1B plasmids in three isolates, while *bla*_SHV-27_ and *bla*_CTX-M-14_/*bla*_CTX-M-15_ were flanked by insertion sequences ISSty2 and ISEc9 on IncFII(K) plasmids, respectively. The iron-scavenging virulence factor Yersiniabactin (encoded by the *ybt* locus), crucial for iron scavenging in *K. pneumoniae*, was mobilized on integrative conjugative elements (ICEKp), which harbored the *virB* operon of the type IV secretion system (T4SS) and the complete *ybt* gene cluster. Seven *ybt* lineages were identified across six ICEKp variants, with the *ybt15* lineage on ICEKp11 being the most prevalent (*n* = 7; five porcine and two duck isolates) (Table S5).

### Genomic and plasmid analysis of KP-HB21H30845

**Fig 3:**
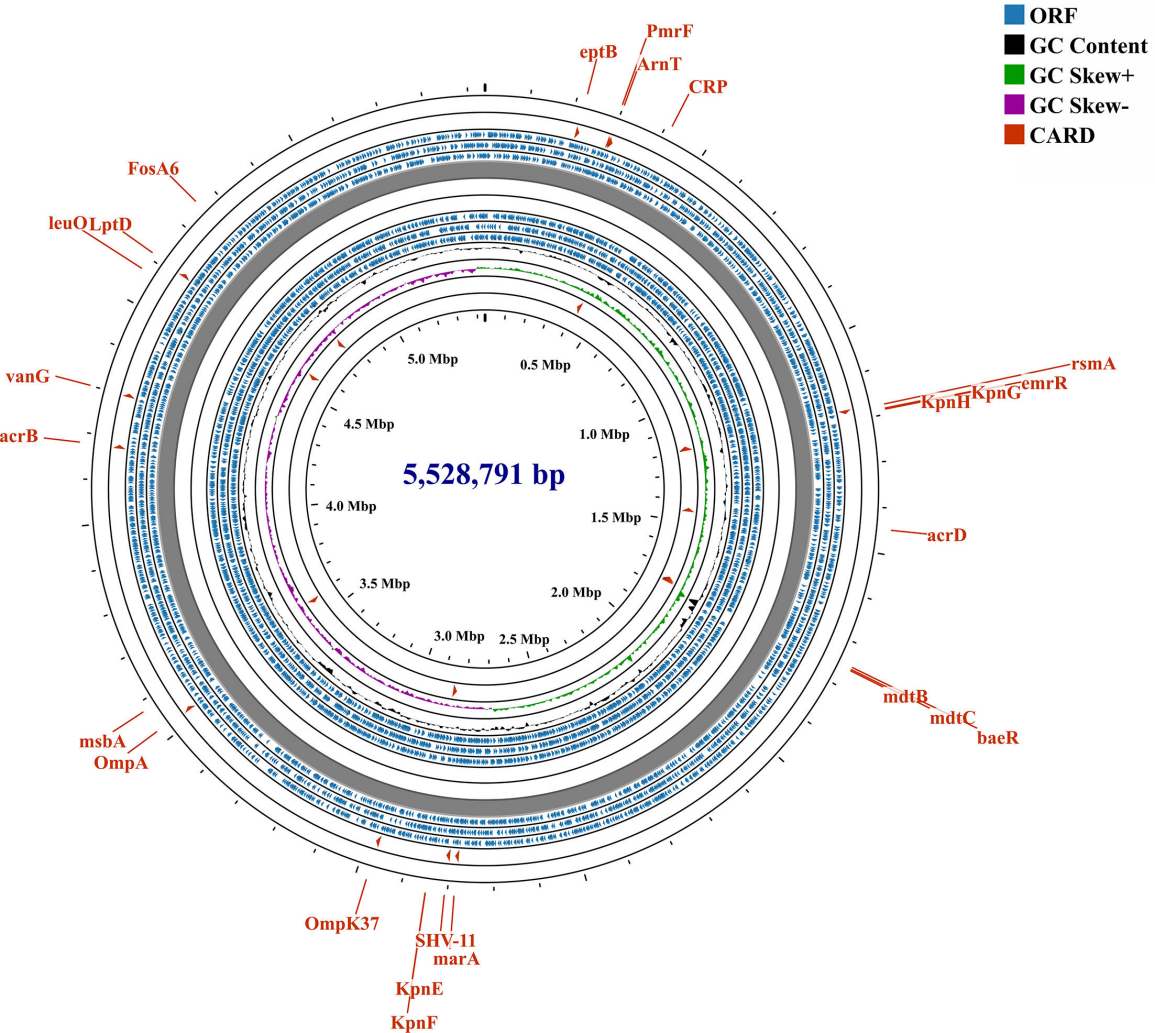
Annotation of the chromosomal genome features in KP-HB21H30845 (accession number: CP188100.1).

To elucidate the genetic basis for the unexpectedly low virulence of KP-HB21H30845 (ST11-KL64), a strain anticipated to exhibit high pathogenicity due to the presence of *iuc* and *rmpA* genes, its genomic and plasmid features were characterized using third-generation sequencing. The genome of KP-HB21H30845 comprises a single chromosome (5,528,791 bp; accession number: CP188100.1) and six plasmids. Chromosomal analysis revealed a repertoire of virulence-associated genes, including adherence factors (*fimA-K* for Type I pili and *mrkA-J* for Type III pili), and iron acquisition systems (*iutA* for aerobactin, *iroB-E* and *iroN* for salmochelin, and *ybtAEPQSTUX*, *irp1*, *irp2*, and *fyuA* for yersiniabactin) (Fig. 3). Notably, the chromosome harbored multiple resistance determinants, including *FosA6* (chromosomally encoded fosfomycin resistance gene), *vanG* (vancomycin resistance), efflux pump genes (*acrBD* and *mdtBC*), and regulatory genes (*ramA*, *soxS*, *marA*, *baeR*, *emrR*, *rsmA*, and *CRP*).

Plasmid analysis using oriTfinder identified six plasmids with distinct functional profiles (Fig. S5). Plasmid 1 (193,822 bp; accession number: CP188101.1) harbored a virulence gene cluster (*iroN*, *rmpA*, and *iucA-D*) and metal resistance genes (*pbrA*, *terA-E*, *terW*, and *terZ*) but lacked the *ybt* locus. Plasmids 2 and 4 contributed to antimicrobial resistance: Plasmid 2 (134,602 bp; accession number: CP188102.1) harbored *bla*_CTX-M-65_, *bla*_KPC-2_, *bla*_SHV-12_, *bla*_TEM-1_, and *rmtB*, alongside metal resistance genes (*sil*, *pco*) and an anti-CRISPR gene (*AcrIE9*). Plasmid 4 (84,870 bp; accession number: CP188104.1) harbored *bla*_LAP-2_, *dfrA14*, *qnrS1*, *sul2*, and *tet(A*). Plasmids 3 (111,727 bp; accession number: CP188103.1), 5 (11,970 bp; accession number: CP188105.1), and 6 (5,596 bp; accession number: CP188106.1) lacked notable functional genes, with Plasmids 5 and 6 exhibiting limited transfer capabilities (*oriC* and *oriT*).

**Fig 4:**
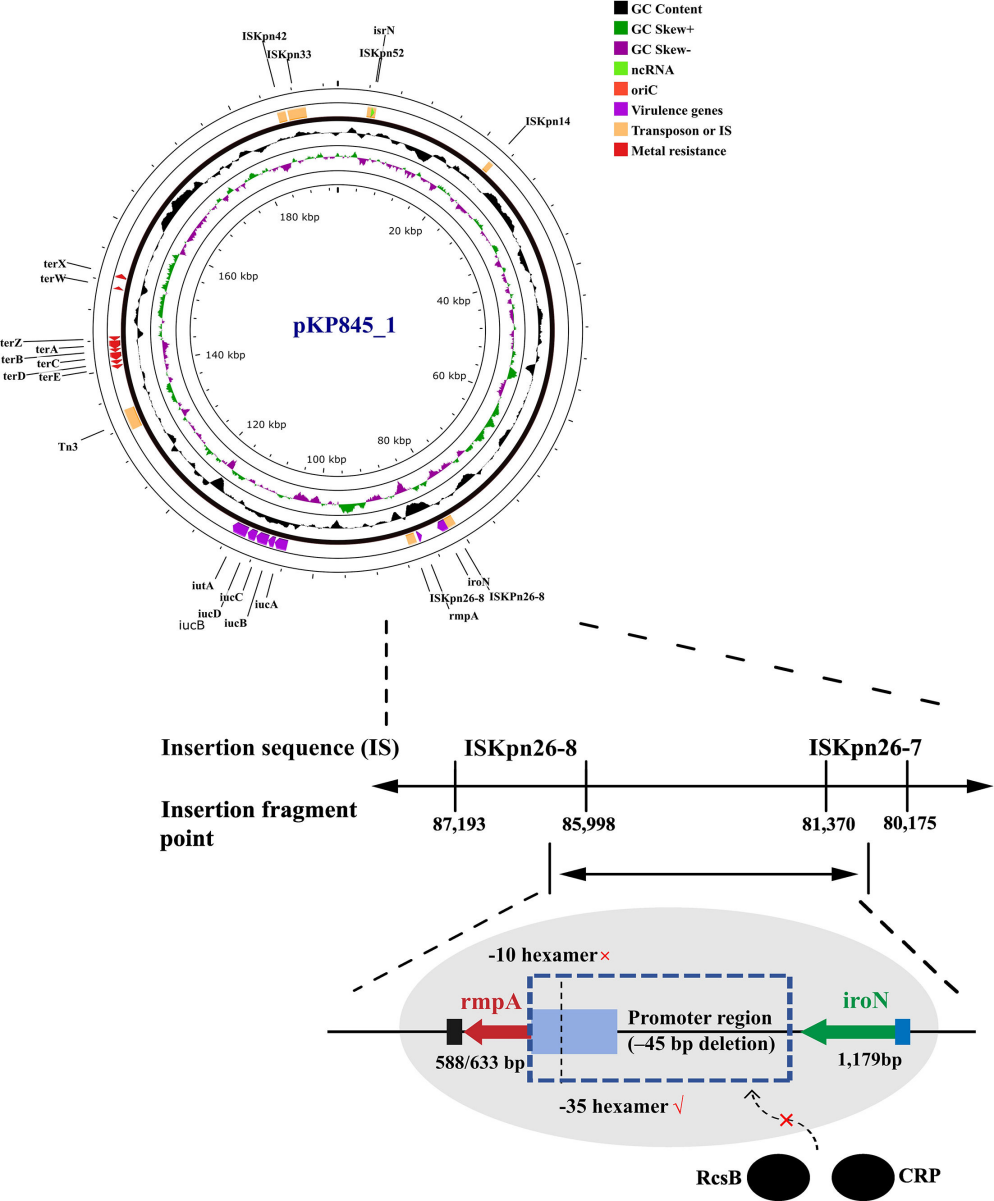
Annotation of pKP845_1 (accession number: CP188101.1). ORFs are portrayed by arrows to indicate the direction of transcription and colored based on their predicted virulence and metal resistance genes functions. The position of the transposon and inserted sequence in the plasmid are highlighted. The *rmpA* gene (85,008–85,595) shows a 45-bp promoter deletion (84,808–84,853) within an ISKpn26 composite transposon, leading to truncation of the –10 hexamer (84,830–84,835) while retaining the –35 hexamer (84,805–84,810).

Sequence analysis of the *rmpA* gene on Plasmid 1 (pKP845_1) revealed a 45 bp deletion in its promoter region (84,808–84,853), likely impairing *rmpA* expression and contributing to reduced virulence (Fig. S6). Located within an ISKpn26-formed composite transposon (80,174–87,193), rmpA (85,008–85,595) is flanked by two ISKpn26 copies (80,175–81,370 and 85,998–87,193), with recombinogenic activity inducing the deletion. The −10 hexamer (84,830–84,835) is truncated, while the −35 hexamer (84,805–84,810) remains intact, potentially disrupting binding of regulators like RcsB and CRP. Additionally, *iroN* (81,370–82,675), adjacent to ISKpn26, may experience transcriptional interference due to its proximity (Fig. 4).

## DISCUSSION

This study provides a comprehensive characterization of AMR profiles and molecular epidemiology of *K. pneumoniae* from human and livestock populations in Central China. This suggests that the continued widespread use of antibiotics in Chinese livestock farming may drive the distinct AMR patterns observed, likely reflecting differences in medication strategies across various breeding systems (20). Specifically, resistance to ceftiofur—a veterinary-specific cephalosporin widely used to treat bovine respiratory and reproductive infections (e.g., pneumonia and metritis)—reached 58.3% in bovine isolates, aligning with its widespread use in cattle husbandry (21). Although plasmid-borne AmpC genes such as CMY-2 were not detected, ESBL determinants were frequently present, which may explain the ceftiofur resistance observed. In contrast, porcine and duck isolates exhibited elevated tetracycline resistance, consistent with their extensive use in swine and poultry farming for growth promotion and enteric infection control (22). The observed meropenem resistance in human isolates may result from its frequent use in intensive care units (ICUs) for treating bacterial infections (23). Interestingly, six isolates lacking *bla*_SHV_ yet resistant to ampicillin suggest alternative β-lactamases or compensatory mechanisms, necessitating hybrid genome assemblies and functional assays to clarify gene loss and clinical impact.

ST101, a recently emerged high-risk clone in hospital settings, harbors numerous antibiotic resistance genes, including carbapenemase-encoding genes, which enhance its adaptive fitness and facilitate global dissemination (24–26). Similarly, ST35 and ST592 have been reported in ICUs-associated outbreaks (27, 28). The emergence of high-risk clones (ST11, ST35, and ST592) in livestock, despite their rarity in animals, indicates potential zoonotic transmission, supported by genetic proximity to human strains and shared AMR profiles. This may involve farmworker contact, environmental contamination, or cross-sector antibiotic pressures, underscoring the need for enhanced surveillance to track transmission routes.

The pathogenicity of *K. pneumoniae* is driven by key virulence factors such as capsular polysaccharides, HMV, and iron acquisition systems (29). *K. pneumoniae* strains displaying the HMV phenotype are associated with increased virulence, commonly attributed to key hypervirulence markers such as *magA* and *rmpA* (30, 31). However, virulence profiling revealed a generally low prevalence of HMV phenotypes, consistent with the absence of *magA* and partial carriage of other key virulence loci (32). Interestingly, most MDR isolates exhibited attenuated virulence in the mmouse lethality assays. This aligns with previous reports of reduced virulence in MDR *K. pneumoniae* and supports the role of *rmpA* as a key hypervirulence marker (33, 34).

In the ST11-KL64 isolate KP-HB21H30845, low virulence was linked to a 45 bp deletion in the *rmpA* promoter on Plasmid 1, located within an ISKpn26-formed composite transposon. This deletion, likely caused by transposon-mediated recombination, impairs *rmpA* expression, abolishing the HMV phenotype. Additionally, *iroN*, adjacent to ISKpn26 within the same transposon, may face transcriptional interference, potentially disrupting iron acquisition and further reducing virulence. Notably, despite chromosomal *ybtAEPQSTUX*, *iutA*, and *iroB-E*, the absence of functional *ybt* on Plasmid 1 highlights plasmid-mediated defects as the primary driver of virulence attenuation (35). This underscores the complex interplay of MGEs and virulence gene expression, indicating that traditional hypervirulence markers may not fully predict pathogenicity in diverse populations. However, short-read sequencing used in this study limits precise plasmid structure resolution, and long-read sequencing or hybrid assemblies are needed to confirm gene localization, a limitation that may affect the accuracy of plasmid-mediated virulence attenuation inferences.

The global emergence of MDR-hypervirulent *K. pneumoniae* (MDR-hvKP) has been increasingly reported (36), driven by MGEs that facilitate the convergence of resistance and virulence (37, 38). In this study, MDR strains harbored large IncF plasmids encoding antibiotic resistance genes and virulence determinants, an evolutionary genetic architecture characteristic of MDR-hvKP (39). Additionally, integrative conjugative elements (ICEs) were identified in several isolates, some of which encoded iron acquisition systems (e.g., *ybt15* on ICEKp11) and efflux pump genes (including *acrAB*-tolC, *oqxAB*, and *emrB*-like transporters), potentially enhancing bacterial fitness during cross-host transmission (40). The limited representation of high-risk clones (e.g., ST35, ST101, ST592) in livestock highlights potential zoonotic risks, necessitating broader genomic surveillance across farming systems. Additionally, functional studies on truncated virulence loci (e.g., *rmpA*) and MGE-mediated gene transfer are essential to quantify the evolutionary risks of MDR-hvKP emergence in veterinary contexts.

### Conclusion

This study elucidates the AMR profiles and molecular epidemiology of *K. pneumoniae* isolates from human and livestock populations in Central China, revealing shared high-risk lineages (ST35, ST101, ST592) and co-localization of resistance and virulence genes on MGEs, which may represent an evolving MDR-hvKP trajectory. The low virulence of the ST11-KL64 strain, attributable to ISKpn26-mediated transcriptional interference of *rmpA* and *iroN*, highlights how virulence defects can attenuate pathogenicity. These findings underscore the need for robust genomic surveillance and control strategies to monitor and prevent potential dissemination of MDR *K. pneumoniae* across human and animal reservoirs.

## MATERIALS AND METHODS

### Bacterial isolates and identification

A total of 42 animal-derived *K. pneumoniae* strains were collected from Hubei and Hunan provinces during 2019–2021, including 24 bovine isolates from 4 farms in Hubei, 11 porcine isolates from 1 farm in Hunan, and 7 duck isolates from 6 farms in Hubei (Table S6). These were supplemented with 10 human clinical isolates obtained from Wuhan University Zhongnan Hospital between January 2020 and June 2021, selected to represent majo STs prevalent in the hospital during this period and isolates exhibiting clinically relevant antimicrobial resistance phenotype, aiming to explore genomic relationships with animal isolates while minimizing temporal and spatial bias. Species identification was confirmed through Gram staining and polymerase chain reaction (PCR) targeting the *khE* and *16S rDNA* genes (41).

### Genomic DNA extraction and whole-genome sequencing

Genomic DNA extraction was performed using the SteadyPure Bacteria Genomic DNA Extraction Kit (Accurate Biotechnology [Hunan, China] Co. Ltd.). Sequencing was conducted on the Illumina NovaSeq PE150 platform via paired-end sequencing. Following quality control, sequencing reads were processed and filtered using FastQC and AdapterRemoval, followed by assembly with SPAdes v3.15.5 (https://github.com/ablab/spades) and annotation with Prokka v1.14.5 (https://github.com/tseemann/prokka). STs were assigned by MLST based on seven conserved housekeeping genes, using the Institut Pasteur MLST database (https://bigsdb.pasteur.fr/). Pathogenwatch analytical tools (https://pathogen.watch/) were employed to predict capsular loci: K-locus (KL) types and *wzi* alleles. AMR genes and virulence factors were identified using ResFinder v4.6.0 and VFDB (VFDB, http://www.mgc.ac.cn/cgi-bin/VFs/v5/main.cgi), respectively, and mapped back to assembled contigs. To infer the chromosomal or plasmid origin of AMR and virulence gene, contigs were classified as plasmid- or chromosome-derived using MOB-suite and mlplasmids. Sequencing quality metrics, including average depth of coverage, genome completeness, and N50 values, were inspected to exclude assembly artifacts. AMR and virulence genes identified by ResFinder and VFDB were mapped back to the classified contigs. Genes located on plasmid-predicted contigs were considered putatively plasmid-borne.

PlasmidFinder v2.0.1 and pMLST v0.1.0 were used to identify plasmid replicons and assign plasmid sequence types. Genes located on contigs carrying plasmid replicons or predicted as plasmid-derived were considered putatively plasmid-borne. Mobile genetic elements were annotated with MGEFinder v1.0.3, and plasmid mobility was predicted with oriTfinder v1.1 to assess transfer potential. Genes located on replicon-free contigs were regarded as likely chromosomal. The MEGA7 software (https://www.megasoftware.net) was used for multiple sequence alignment of the *rmpA* gene and visualized using Mview v1.63 (https://desmid.github.io/mview/).

### Phylogenetic tree construction

A total of 107 complete genome sequences of *K. pneumoniae* strains (ST11, ST35, ST101, and ST592), including 94 human-derived and 13 animal-derived isolates, were retrieved from the NCBI database (Table S2). A core-genome alignment was generated using Parsnp v1.2, with the complete genome of *K. pneumoniae* strain HS11286 (accession number: CP003200.1) as the reference genome. SNP distances were inferred from the resulting XMFA alignment. The phylogenetic tree was visualized using iTOL v7 (https://itol.embl.de/). A core genome multilocus sequence typing (cgMLST) analysis was conducted using SeqSphere + v10.0 (Ridom GmbH, Münster, Germany) based on the *K. pneumoniae* cgMLST scheme (2,358 loci). Allele-based minimum spanning trees (MSTs) were constructed to visualize phylogenetic relatedness among isolates from different hosts and geographic sources.

### Determination of antimicrobial resistance

Antimicrobial susceptibility profiles of the isolates were determined by the broth microdilution method in accordance with the guidelines of the Clinical and Laboratory Standards Institute (CLSI). Human clinical isolates were tested according to CLSI M100-Ed35 (2025) (42), whereas animal-derived isolates conformed to CLSI VET09-Ed2 (2024) criteria (43). Susceptibility testing included 17 antimicrobial agents representing various classes: β-lactams (ampicillin [AMP], amoxicillin/clavulanic acid [AMC], cephalothin [CEP], ceftiofur [CEF], ceftriaxone [CRO], and meropenem [MEM]), aminoglycosides (streptomycin [STR], kanamycin [KAN], and gentamicin [GEN]), tetracyclines (tetracycline [TET] and doxycycline [DOX]), fluoroquinolones (ciprofloxacin [CIP]), folate pathway inhibitors (sulfamethoxazole/trimethoprim [SXT] and sulfisoxazole [SOX]), and miscellaneous agents (colistin [CT], rifaximin [RFX], and florfenicol [FFC]). Susceptibility interpretive criteria were derived from CLSI M100-Ed35 and CLSI VET09-Ed2 breakpoint guidelines, using *K. pneumoniae* ATCC 700603 as the quality control strain. MDR strains were defined as isolates exhibiting resistance to at least three distinct antibiotic classes, in line with internationally accepted MDR classification criteria. The ESBL phenotype was confirmed via broth microdilution-based clavulanic acid potentiation assays, while carbapenemase production in meropenem-resistant strains was screened using the modified carbapenem inactivation method (mCIM), both conducted according to CLSI M100-Ed35 (2025) guidelines (42).

### String test

The HMV phenotype was tested using the string test. *K. pneumoniae* strains were inoculated onto MacConkey agar plates and incubated overnight at 37°C. A positive HMV phenotype was defined as the formation of an adhesive string exceeding 5 mm in length when a colony was gently lifted with a sterile inoculation loop (44).

### Mouse lethality test

Based on MLST and string test results, nine strains exhibiting HMV and high-virulence characteristics were selected for the mouse lethality test. A total of 384 specific pathogen-free (SPF) male BALB/c mice (6 weeks old; 15–18 g; 6 mice/cage) were housed under controlled conditions (22 ± 1°C, 55% ± 5% humidity, a 12 h light/dark cycle) with *ad libitum* access to sterile food and water. After a 7-day acclimation period, bacterial cultures were grown to the logarithmic phase (OD_600_ = 0.6–0.8), washed twice with sterile saline, and resuspended to a final concentration of 10^3^–10^9^ CFU/mL. Six mice in each concentration group were intraperitoneally administered 100 µL of bacterial suspension, while control mice received an equivalent volume of sterile saline. Survival rates, clinical signs and symptoms, and mortality were monitored daily for 7 days. The median lethal dose (LD₅₀) was calculated using the probit analysis method in SPSS Statistics v26 , with virulence interpretation referenced to established criteria (45).

### Statistical analyses

Statistical analyses were performed using SPSS Statistics v26 (IBM, Armonk, NY, USA) and GraphPad Prism software v5.01 (GraphPad Software, San Diego, CA, USA). Pearson’s correlation coefficients were calculated to assess relationships between variables. A *P*-value < 0.05 is considered statistically significant.

## Data Availability

The complete genomes of 52 *K*. *pneumoniae* isolates were deposited in the NCBI database (Bioproject accessions: PRJNA831618, PRJNA831619, PRJNA831621, PRJNA832498, PRJNA839518, PRJNA832529, PRJNA832530, PRJNA832895, PRJNA832918, PRJNA832923, PRJNA832924, PRJNA832927, PRJNA832934, PRJNA832936, and PRJNA1148939).

## SUPPLEMENTAL MATERIAL

Supplemental material spectrum.02269-25-s0001.pdf Fig. S1-S6; Tables S1-S6.

ASM does not own the copyrights to Supplemental Material that may be linked to, or accessed through, an article. The authors have granted ASM a non-exclusive, world-wide license to publish the Supplemental Material files. Please contact the corresponding author directly for reuse.
