# Supplementary material for: Antimicrobial resistance, virulence defects, and evolutionary dynamics of multidrug-resistant Klebsiella pneumoniae from human and animal hosts in Central China
Source: Microbiol Spectr. 2025 Dec 17;14(2):e02269-25. doi: 10.1128/spectrum.02269-25 (PMC12889024; doi:10.1128/spectrum.02269-25)
Supplement: Supplemental material — Fig. S1-S6; Tables S1-S6. [file spectrum.02269-25-s0001.pdf]

Supplementary Material

Antimicrobial Resistance, Virulence Defects, and Evolutionary Dynamics of Multidrug-Resistant *Klebsiella pneumoniae* from Human and Animal Hosts in Central China

Wen Sun[1], Xiaoman Zhu[1], Xiaomin Pang[1], Xiangyun Wu[1,3], Qian Guo[1], Linshen Li[1], Baojing Dou[1], Yulian Wang[1,2*]

[1] National Reference Laboratory of Veterinary Drug Residues and MOA Key Laboratory for Detection of Veterinary Drug Residues, Huazhong Agricultural University, Wuhan 430070, China.

[2] MOA Laboratory for Risk Assessment of Quality and Safety of Livestock and Poultry Products, Huazhong Agricultural University, Wuhan 430070, China.

[3] Anlu Animal Disease Prevention and Control Center, Anlu 432600, China.

*** Correspondence:**

Yulian Wang (Y. Wang) , Mail address: National Reference Laboratory of Veterinary Drug Residues, Huazhong Agricultural University, Wuhan 430070, China.

E-mail: wangyulian@mail.hzau.edu.cn

Figures

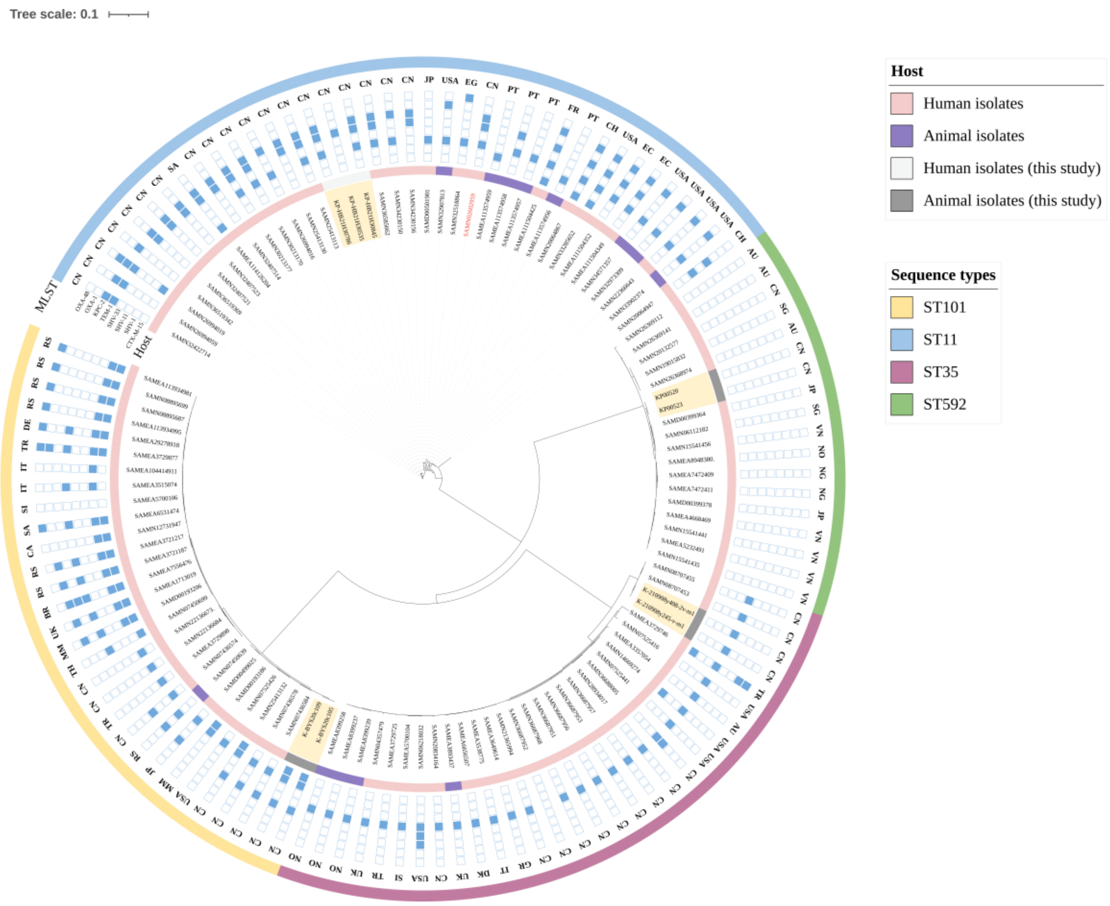

Figure S1. Phylogenetic tree of *K. pneumoniae* sequence types ST11, ST35, ST101, and ST529 based on SNP loci. The ST11, ST35, ST101, and ST529 isolates in this study have an orange background color, while the reference strain is marked in red. CN, China; RS, Serbia; MM, Myanmar; IT, Italy; TR, Turkey; TH, Thailand; UK, United Kingdom; SA, Saudi Arabia; SI, Slovenia; USA, United States of America; DE, Germany; CA, Canada; BR, Brazil; JP, Japan; CH, Switzerland; FR, France; EC, Ecuador; EG, Egypt; PT, Portugal; GR, Greece; AU, Australia; DK, Denmark; NO, Norway; VN, Vietnam; NG, Nigeria; SG, Singapore

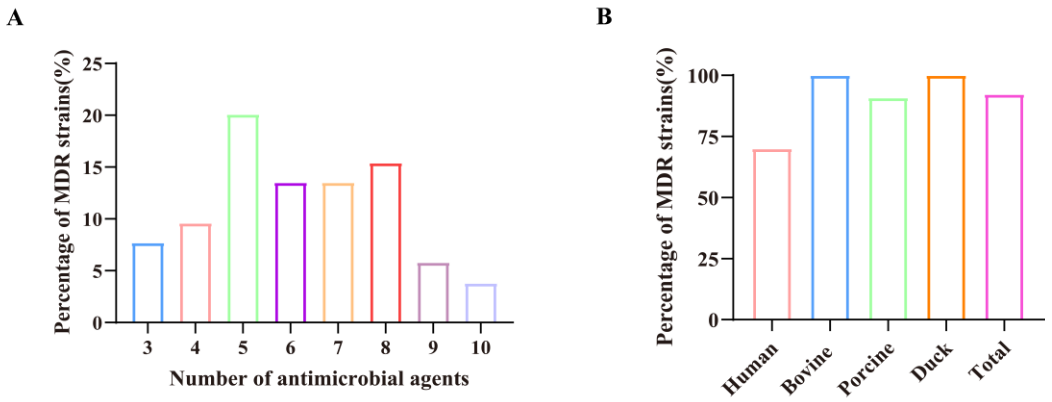

Figure S2. Epidemiological characteristics of MDR in *K. pneumoniae*. A: Percentages of *K. pneumoniae* isolates resistant to different numbers of antibiotics. B: Percentages of MDR *K. pneumoniae* among isolates from different sources.

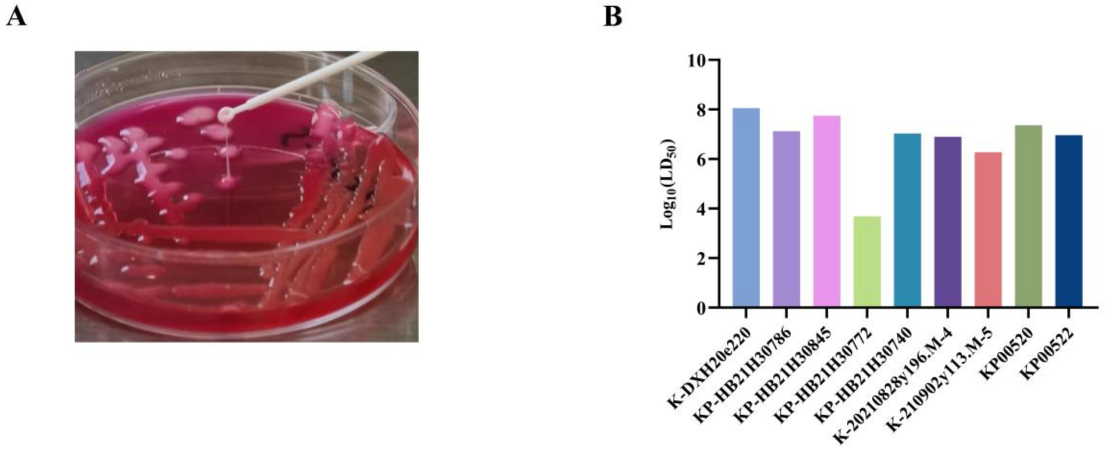

Figure S3. In vitro and in vivo virulence phenotypes of *K. pneumoniae*. A: Colony morphology of hypermucoviscous *K. pneumoniae*. B: Mouse lethality test for in vivo virulence. Nine representative isolates (selected based on ST, resistance profile, or colony phenotype) were tested in a BALB/c mouse model.

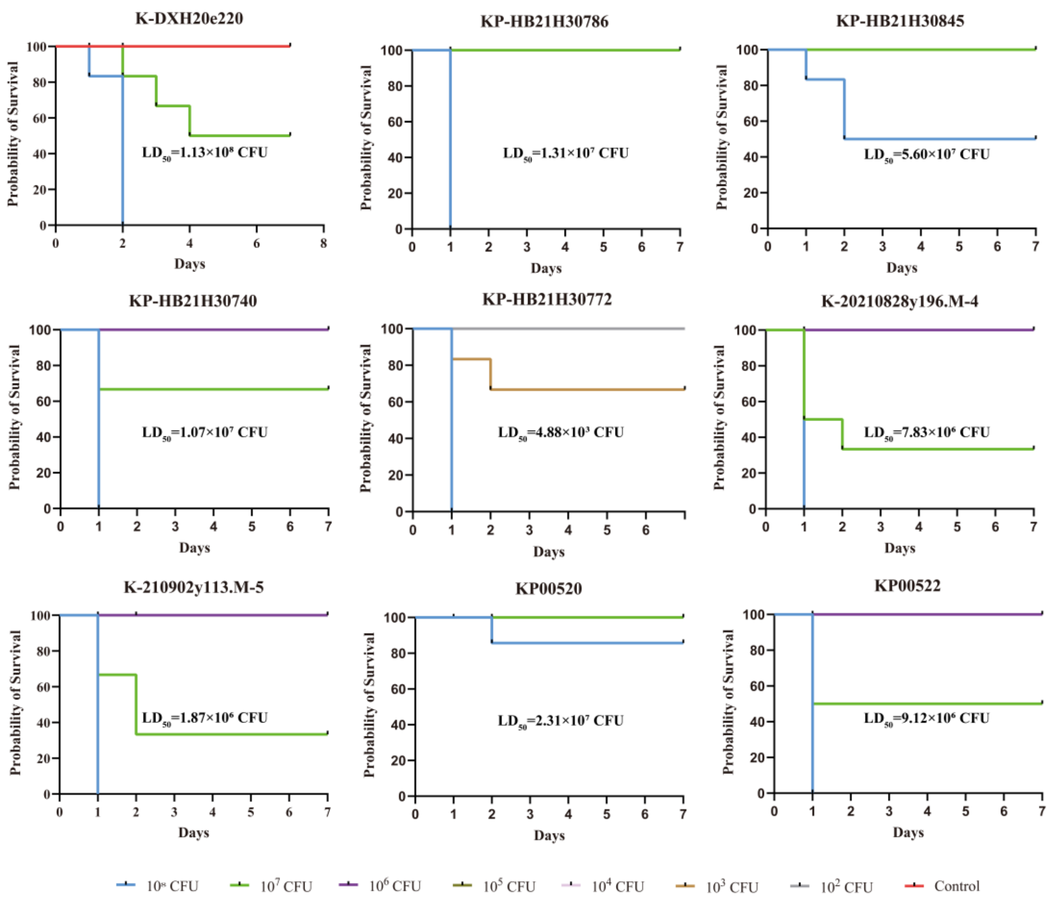

Figure S4. Survival curves of mice inoculated with *K. pneumoniae*. Mice with different inoculation doses of intraperitoneal injection are represented by different colored lines.

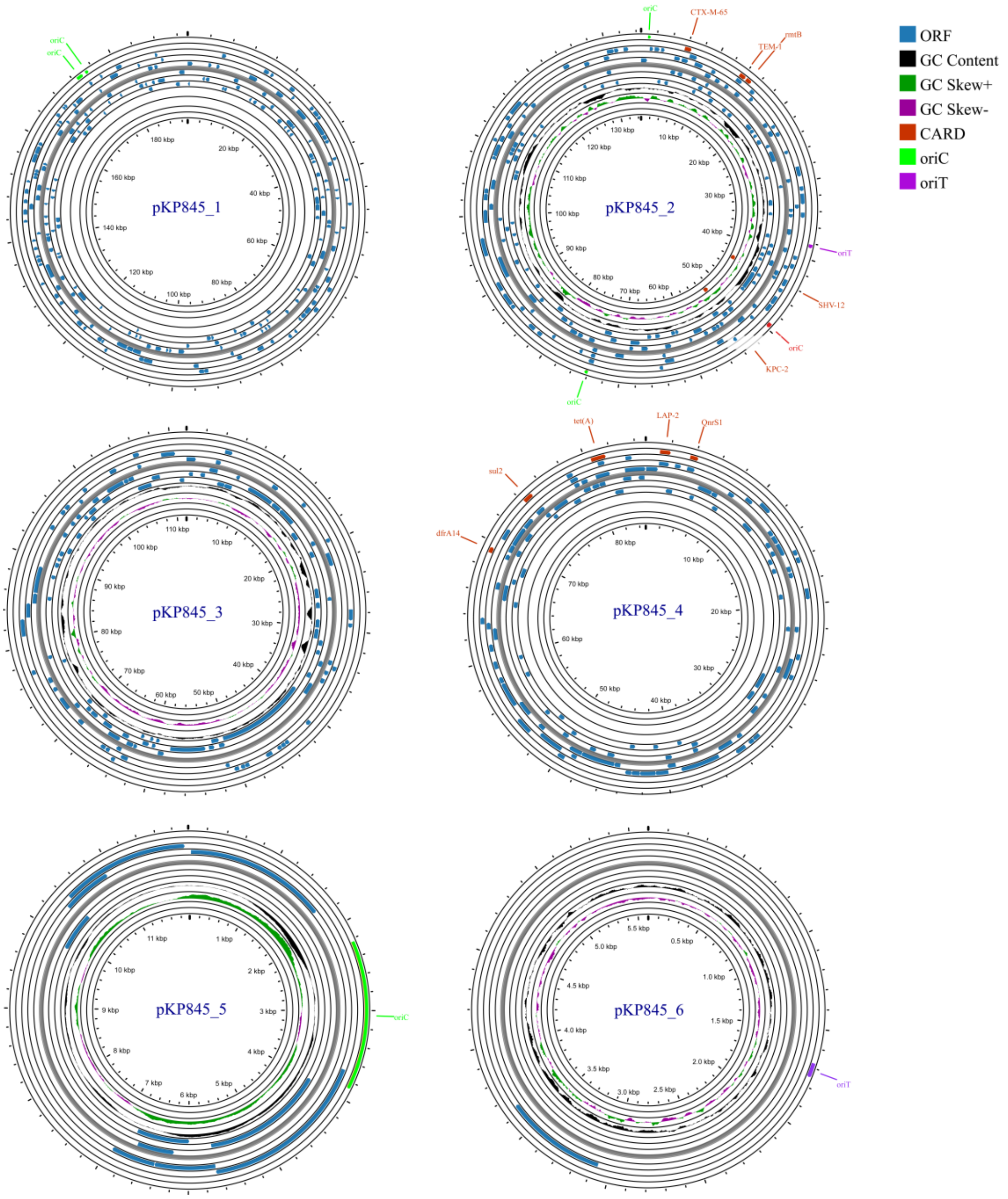

Figure S5. Annotation of the plasmid genome features and resistance genes in KP-HB21H30845 (pKP845_1 accession number: CP188101.1; pKP845_2 accession number: CP188102.1; pKP845_3 accession number: CP188103.1; pKP845_4 accession number: CP188104.1; pKP845_5 accession number: CP188105.1; pKP845_6 accession number: CP188106.1).

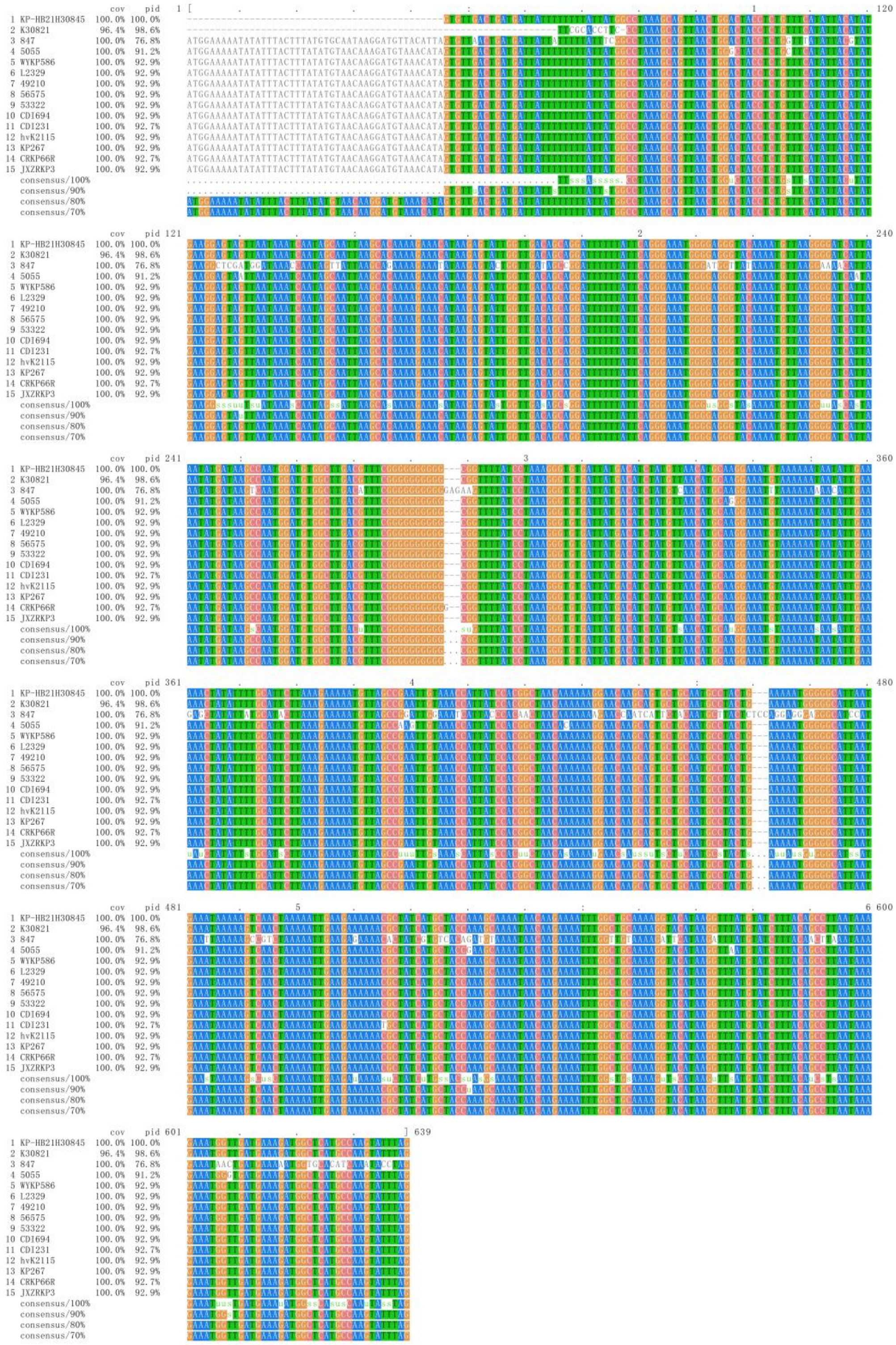

Figure S6. Results of CDS sequence alignment of the *rmpA* gene of *K. pneumoniae* (K30821 accession number: NZ_CP107015.1; 847 accession number: AY059956.1; 5055 accession number: AY059958.1; WYKP586 accession number: OQ801413.1; L2329 accession number: CP136844.1;

49210 accession number: CP089030.1; 56575 accession number: CP089013.1; 53322 accession number: CP088990.1; CDI694: CP077778.1; CDI231 accession number: CP077784.1; hvk2115 accession number: CP091327.1; KP267 accession number: MG053312.1; CRKP66R accession number: NZ_CP063834.1; JXZRKP3 accession number: CP174262.1).

Tables

Table S1. Characteristics of the genomes of 52 *K. pneumoniae* strains.

Strains	Origins	Sample types	Regions	MLST	*wzi* alleles	K-locus types	Biosample	HMV phenotype
K-BYS20c105	Bovine	Nipple skin swab of CM cow	Hubei	101	*wzi*-29	KL106	SAMN27755378	No
K-BYS20c109	Bovine	Nipple skin swab of CM cow	Hubei	101	*wzi*-29	KL106	SAMN27755381	No
K-MJD20e150	Bovine	Nipple milk of CM cow	Hubei	5855	*wzi*-546	KL107	SAMN27755385	No
K-MJD20c164	Bovine	Nipple skin swab of SCM cow	Hubei	1787	*wzi*-325	KL148	SAMN27863983	No
K-MJD20b201	Bovine	Anal swab of CM cow	Hubei	36	*wzi*-173	KL102	SAMN28535392	No
K-DXH20e220	Bovine	Nipple milk of SCM cow	Hubei	5874	*wzi*-502	KL107	SAMN27865097	No
K-DXH20e225	Bovine	Nipple milk of CM cow	Hubei	1037	*wzi*-243	KL7	SAMN27865099	No
K-DXH20e226	Bovine	Nipple milk of CM cow	Hubei	1537	*wzi*-64	KL64	SAMN27924902	No
K-DXH20e227	Bovine	Nipple milk of CM cow	Hubei	4452	*wzi*-23	KL36	SAMN27925674	No
K-DXH20e228	Bovine	Nipple milk of CM cow	Hubei	4452	*wzi*-23	KL36	SAMN27925759	No
K-DXH20e230	Bovine	Nipple milk of CM cow	Hubei	234	*wzi*-272	KL30	SAMN27925760	No
K-DXH20e231	Bovine	Nipple milk of CM cow	Hubei	661	*wzi*-209	KL47	SAMN27925788	No
K-DXH20e232	Bovine	Nipple milk of CM cow	Hubei	234	*wzi*-454	KL30	SAMN27925892	No

K-DXH20e233	Bovine	Nipple milk of CM cow	Hubei	5875	*wzi*-81	KL120	SAMN27925917	No
K-JX19e03	Bovine	Nipple milk of CM cow	Hubei	2854	*wzi*-150	KL183	SAMN43225281	No
K-MJD19e39	Bovine	Nipple milk of CM cow	Hubei	5370	*wzi*-150	KL40	SAMN43225282	No
K-MJD19e29	Bovine	Nipple milk of CM cow	Hubei	2854	*wzi*-150	KL183	SAMN43225283	No
K-MJD19e25	Bovine	Nipple milk of CM cow	Hubei	5370	*wzi*-150	KL40	SAMN43225284	No
K-MJD19e35	Bovine	Nipple milk of CM cow	Hubei	2854	*wzi*-150	KL183	SAMN43225285	No
K-MJD19e32	Bovine	Nipple milk of CM cow	Hubei	5370	*wzi*-150	KL40	SAMN43225286	No
K-BYS20e89	Bovine	Nipple milk of CM cow	Hubei	528	*wzi*-27	KL27	SAMN43225287	No
K-MJD20e148	Bovine	Nipple milk of CM cow	Hubei	5915	*wzi*-375	KL142	SAMN43225288	No
K-MJD20e149	Bovine	Nipple milk of CM cow	Hubei	5915	*wzi*-376	KL142	SAMN43225289	No
K-DXH20e234	Bovine	Nipple milk of CM cow	Hubei	219	*wzi*-122	KL121	SAMN43225290	No
KP-HB21H30786	Human	Blood sample of patient	Hubei	11	*wzi*-64	KL64	SAMN43225291	No
KP-HB21H30727	Human	Blood sample of patient	Hubei	15	*wzi*-19	KL19	SAMN43225292	No
KP-HB21H30799	Human	Blood sample of patient	Hubei	152	*wzi*-124	KL105	SAMN43225293	No
KP-HB21H30845	Human	Blood sample of patient	Hubei	11	*wzi*-64	KL64	SAMN43225294	No
KP-HB21H30826	Human	Blood sample of patient	Hubei	727	*wzi*-83	KL23	SAMN43225295	No
KP-HB21H30761	Human	Blood sample of patient	Hubei	721	*wzi*-25	KL25	SAMN43225296	No

KP-HB21H30535	Human	Blood sample of patient	Hubei	11	*wzi*-64	KL64	SAMN43225297	Yes
KP-HB21H30813	Human	Blood sample of patient	Hubei	1419	*wzi*-123	KL136	SAMN43225298	No
KP-HB21H30772	Human	Blood sample of patient	Hubei	86	*wzi*-2	KL2	SAMN43225299	Yes
KP-HB21H30740	Human	Blood sample of patient	Hubei	2410	*wzi*-274	KL30	SAMN43225300	No
K-210828y126.V-2	Porcine	Vaginal swab of diseased pig	Hunan	2410	*wzi*-274	KL30	SAMN43225301	No
K-20210828y126.M-2	Porcine	Nipple milk of diseased pig	Hunan	2410	*wzi*-274	KL30	SAMN43225302	No
K-20210828y196.M-1	Porcine	Nipple milk of diseased pig	Hunan	2410	*wzi*-274	KL30	SAMN43225303	No
K-20210828y196.M-4	Porcine	Nipple milk of diseased pig	Hunan	2410	*wzi*-274	KL30	SAMN43225304	Yes
K-210830y115.M-5	Porcine	Nipple milk of diseased pig	Hunan	2410	*wzi*-274	KL30	SAMN43225305	Yes
K-210902y113.M-5	Porcine	Nipple milk of diseased pig	Hunan	2410	*wzi*-274	KL30	SAMN43225306	No
K-210908y245-v-m1	Porcine	Vaginal swab of diseased pig	Hunan	35	*wzi*-194	KL108	SAMN43225307	No
K-210908y408-2v-m1	Porcine	Vaginal swab of diseased pig	Hunan	35	*wzi*-194	KL108	SAMN43225308	No
K-211002y45204V-2-M	Porcine	Vaginal swab of diseased pig	Hunan	2459	*wzi*-596	KL125	SAMN43225309	No
K-211002y64V-1-M	Porcine	Vaginal swab of diseased pig	Hunan	6431	*wzi*-295	KL49	SAMN43225310	No
KQ-211002y64V-2-M	Porcine	Vaginal swab of diseased pig	Hunan	6432	*wzi*-730	KL127	SAMN43225311	No
KP00519	Duck	Lung sample of diseased duck	Hubei	2410	*wzi*-274	KL30	SAMN43225312	No
KP00520	Duck	Lung sample of diseased duck	Hubei	592	*wzi*-206	KL57	SAMN43225313	No

KP00521	Duck	Lung sample of diseased duck	Hubei	6082	*wzi*-130	KL58	SAMN43225314	No
KP00522	Duck	Lung sample of diseased duck	Hubei	2410	*wzi*-274	KL30	SAMN43225315	No
KP00523	Duck	Lung sample of diseased duck	Hubei	592	*wzi*-206	KL57	SAMN43225316	No
KP00524	Duck	Lung sample of diseased duck	Hubei	111	*wzi*-73	KL104	SAMN43225317	No
KP00525	Duck	Lung sample of diseased duck	Hubei	6082	*wzi*-130	KL58	SAMN43225318	Yes

Table S2. Metadata of international *K. pneumoniae* strains from ST11, ST35, ST101, and ST592 lineages in the NCBI database.

BioSample	MLST	Locations	Hosts
SAMN08895687	101	Serbia	Human
SAMD00193206	101	Myanmar	Human
SAMD00193186	101	Myanmar	Human
SAMEA3515074	101	Italy	Human
SAMEA3729877	101	Turkey	Human
SAMN07450699	101	Thailand	Human
SAMEA1713019	101	United Kingdom	Human
SAMEA6531474	101	Saudi Arabia	Human
SAMEA5700106	101	Slovenia	Human
SAMN07525426	101	United States of America	Human
SAMN08895699	101	Serbia	Human
SAMEA3729898	101	Turkey	Human
SAMEA3721217	101	Serbia	Human
SAMEA104414911	101	Italy	Human
SAMEA29278918	101	Germany	Human
SAMEA3721187	101	Serbia	Human
SAMN12731947	101	Canada	Human
SAMEA7556476	101	Brazil	Human
SAMN07436574	101	China	Human
SAMN07436578	101	China	Human
SAMN07436584	101	China	Human
SAMN22136673	101	China	Human
SAMN22136684	101	China	Human
SAMN25413132	101	China	Human
SAMD00499025	101	Japan	Human
SAMEA113934995	101	Serbia	Human

SAMEA113934981	101	Serbia	Human
SAMN07450639	101	Serbia	Animals
SAMN02602959	11	China	Human
SAMN20064947	11	Switzerland	Human
SAMN20064867	11	Switzerland	Human
SAMN22366643	11	United States of America	Human
SAMN25413130	11	China	Human
SAMN26994019	11	China	Human
SAMN25413113	11	China	Human
SAMN32407523	11	China	Human
SAMN30213177	11	China	Human
SAMN26994059	11	China	Human
SAMN26994016	11	China	Human
SAMN32422714	11	China	Human
SAMEA111504425	11	France	Human
SAMEA111504349	11	Ecuador	Human
SAMEA111504352	11	Ecuador	Human
SAMD00501901	11	Japan	Human
SAMEA114126204	11	Saudi Arabia	Human
SAMN32518864	11	Egypt	Human
SAMN33285652	11	United States of America	Human
SAMN32973309	11	United States of America	Animals
SAMEA113574957	11	Portugal	Animals
SAMN34571357	11	United States of America	Animals
SAMEA113574956	11	Portugal	Animals
SAMEA113574959	11	Portugal	Animals
SAMEA113574958	11	Portugal	Animals
SAMN32907813	11	United States of America	Animals
SAMN33902374	11	United States of America	Animals

SAMN30213170	11	China	Human
SAMN32407514	11	China	Human
SAMN32407521	11	China	Human
SAMN34230150	11	China	Human
SAMN34230156	11	China	Human
SAMN36519342	11	China	Human
SAMN36519369	11	China	Human
SAMN36585662	11	China	Human
SAMEA3893437	35	United Kingdom	Animals
SAMEA5700104	35	Slovenia	Human
SAMEA3729746	35	Turkey	Human
SAMEA3729725	35	Turkey	Human
SAMEA3538775	35	Italy	Human
SAMEA3649614	35	Greece	Human
SAMN04357479	35	United Kingdom	Human
SAMN06218032	35	United States of America	Human
SAMN14669274	35	United States of America	Human
SAMN07525441	35	United States of America	Human
SAMN07525416	35	United States of America	Human
SAMN08707453	35	China	Human
SAMN08707455	35	China	Human
SAMEA3357054	35	Australia	Human
SAMEA6656507	35	Denmark	Human
SAMN20834164	35	China	Human
SAMN21365994	35	China	Human
SAMN36687953	35	China	Human
SAMN36687956	35	China	Human
SAMN36687952	35	China	Human
SAMN36687968	35	China	Human

SAMN28934017	35	China	Human
SAMN36687957	35	China	Human
SAMN36688005	35	China	Human
SAMN36687951	35	China	Human
SAMEA8399237	35	Norway	Animals
SAMEA8399258	35	Norway	Animals
SAMEA8399239	35	Norway	Animals
SAMD00399364	592	Japan	Human
SAMD00399378	592	Japan	Human
SAMEA4668469	592	Vietnam	Human
SAMEA5232491	592	Vietnam	Human
SAMEA7472409	592	Nigeria	Human
SAMEA7472411	592	Nigeria	Human
SAMEA8948380	592	Norway	Human
SAMN06112182	592	Singapore	Human
SAMN15541435	592	Vietnam	Human
SAMN15541441	592	Vietnam	Human
SAMN15541456	592	Vietnam	Human
SAMN19015832	592	Singapore	Human
SAMN20132577	592	China	Human
SAMN26368974	592	Australia	Human
SAMN26369112	592	Australia	Human
SAMN26369141	592	Australia	Human

Table S3. Distribution of major β-lactam resistance genes in 52 *K. pneumoniae* from different host origins.

**Strain origins**	**Distribution of β-lactam resistance genes % (number of isolates)**				
	ESBL phenotype	bla_{SHV}	bla_{KPC}	bla_{TEM}	bla_{CTX-M}
Bovine (24)	58.3 (14)	83.3 (20)	0.0 (0)	58.3 (14)	54.2 (13)
Human (10)	70.0 (7)	100.0 (10)	40.0 (4)	50.0 (5)	40.0 (4)
Porcine (11)	0.0 (0)	81.8 (9)	0.0 (0)	9.1 (1)	0.0 (0)
Duck (7)	0.0 (0)	100.0 (7)	0.0 (0)	28.6 (2)	0.0 (0)
Total (52)	40.4 (21)	88.5 (46)	7.7 (4)	42.3 (22)	32.7 (17)

Table S4. Association of mobile genetic elements (MGEs) with antimicrobial resistance genes (ARGs) and virulence genes (VFGs) of 52 *K. pneumoniae* strains.

**Strains**	**plasmid multilocus sequence typings**	**Plasmids replicons**	**Insertion sequences**	**Unit transposons**	**Resistance genes**	**Virulence genes**
K-BYS20c105	IncF [K12:A-:B-]	/	ISEc9	/	*bla*$_{CTX-M-15}$	*traT*
		/	IS102	/	/	*clpK1*
		/	/	Tn5403	*tet*(A), *qnrB1*	/
		/	ISEcl1	/	/	/
		/	ISEc52	/	/	/
		/	ISKpn1	/	/	/
		/	IS5075	/	/	/
		/	ISSty2	/	/	/
		/	IS26	/	/	/
K-BYS20c109	IncF [K12:A-:B-]	IncFIB(K)	/	/	/	/
		/	ISEc9	/	*bla*$_{CTX-M-15}$	t*raT*
		/	IS102	/	/	*clpK1*
		/	/	Tn5403	*tet*(A), *qnrB1*	/
		/	ISEcl1	/	/	/
		/	ISEc52	/	/	/
		/	ISKpn1	/	/	/
		/	IS5075	/	/	/
		/	ISSty2	/	/	/
		/	IS26	/	/	/
K-MJD20e150	IncF [F-:A-:B-]	IncFIA(HI1)	/	/	/	/
		Col440I	/	/	/	/
		IncR	/	/	/	/
		/	ISKpn1	/	/	/

		/	IS903, IS26	/	/	/
K-MJD20c164	IncF [F-:A-:B-]	IncFIB(K)	ISKox1	/	/	/
		/	ISEc9	/	*bla*$_{TEM-1B}$, *sul2*, *aph(6)-Id*, *bla*$_{CTX-M-15}$, *aph(3'')-Ib*	/
		/	IS6100	/	*dfrA14*	/
		/	ISSty2	/	*bla*$_{SHV-172}$	*irp2*, *fyuA*
		/		Tn5403	*qnrB1*	/
		/	ISEam1, ISKpn14	/	/	/
		/	ISKpn2	/	/	/
		/	ISEc33	/	/	/
		/	ISEch12	/	/	/
		/	ISKpn24	/	/	/
		/	ISKpn1	/	/	/
		/	IS5075	/	/	/
K-MJD20b201	IncF [K9:A-:B-]	IncFIB(Mar)	/	/	/	/
		IncFIB(K)	/	/	/	/
		IncFII	/	/	/	/
		IncFII(K)	/	/	/	/
		/	ISEc9	/	*bla*$_{CTX-M-14}$, *OqxB*, *OqxA*	/
		/	ISKpn24	/	/	*terC*
		/	ISKpn41	/	/	/
		/	ISKpn21, ISKpn38, ISEsa1, ISKpn28	/	/	/

		/	ISEc52	/	/	/
		/	IS903	/	/	/
		/	ISKpn26	/	/	/
		/	ISKpn47	/	/	/
		/	ISKpn8	/	/	/
		/	ISKpn1	/	/	/
K-DXH20e220	IncF [K2:A-:B-]	IncFIB(K)	ISKox1	/	/	/
		IncQ1	/	/	*aph(6)-Id*, *aph(3'')-Ib*, *sul2*	/
		IncFII(K)	/	/	/	/
		Col440I	/	/	/	/
		/	ISKpn47	/	/	*traT*
		/	ISKpn2	/	/	/
		/	IS5075	/	/	/
		/	ISSen3	/	/	/
		/	ISSen4	/	/	/
		/	ISKpn28	/	/	/
		/	ISEhe3	/	/	/
		/	ISKpn21	/	/	/
		/	ISKpn1	/	/	/
		/	ISKpn42	/	/	/
		/	IS26	/	/	/
K-DXH20e225	IncF [F-:A-:B-]	IncHI1B	IS5075	/	*tet*(A)	/
		IncFIB(K)	ISKox1, ISKpn42	/	/	/
		Col440I	/	/	/	/

		/	ISSty2	/	*bla*$_{SHV-40}$, *bla*$_{SHV-85}$, *bla*$_{SHV-79}$, *bla*$_{SHV-56}$, *bla*$_{SHV-89}$	/
		/	ISKpn1, ISEam1	/	/	/
		/	ISKpn21	/	/	/
		/	ISSen4	/	/	/
		/	ISEhe3	/	/	/
K-DXH20e226	IncF [F-:A-:B-]	IncFIB(K)	ISKox1, ISKpn42	/	/	/
		IncHI1B	/	/	/	/
		Col440I	/	/	/	/
		/	ISSen3, ISEhe3	/	/	/
		/	ISKpn26	/	/	/
		/	ISKpn21	/	/	/
		/	ISKpn38	/	/	/
		/	ISKpn24	/	/	/
		/	ISKpn19	/	/	/
K-DXH20e227	IncF [K2:A-:B-]	IncFIB(K)	ISKpn2	/	/	/
		ColRNAI	/	/	/	*ccI*
		IncFII(K)	/	/	/	/
		/	ISKpn19	/	*qnrS1*	/
		/	/	GIE492	/	*mchF*
		/	ISKpn47	/	/	*traT*
		/	IS5075	/	/	/
		/	ISSen4	/	/	/

		/	ISKpn42	/	/	/
		/	ISKpn21	/	/	/
		/	ISSen3, ISEhe3	/	/	/
		/	ISKpn1	/	/	/
K-DXH20e228	IncF [K2:A-:B-]	IncFIB(K)	ISKpn2	/	/	/
		ColRNAI	/	/	/	*ccI*
		IncFII(K)	/	/	/	/
		/	/	GIE492	/	*mchF*
		/	ISKox1	/	/	/
		/	ISKpn47	/	/	/
		/	IS5075	/	/	/
		/	ISSen4	/	/	/
		/	ISSen3	/	/	/
		/	ISKpn21	/	/	/
		/	ISKpn42	/	/	/
		/	ISKpn1	/	/	/
K-DXH20e230	IncF [F-:A-:B-]	IncHI1B	ISKpn41, IS5075, IS102	/	*tet*(A)	/
		IncFIB(Mar)	/	/	/	/
		/	ISKpn21	/	/	*terC*
		/	ISKpn26	/	/	/
		/	ISKpn34	/	/	/
		/	IS5	/	/	/
K-DXH20e231	IncF [K2:A-:B-]	IncFIB(K)	ISKox1	/	/	/
		IncFII(K)	/	/	/	/
		/	ISEcl10	/	*OqxB*, *OqxA*	*fimH*, *mrkA*

		/	ISKpn21	/	*bla*$_{SHV-27}$	/
		/	IS5075	/	/	/
		/	ISSen3	/	/	/
		/	ISKpn47	/	/	/
		/	ISSen4	/	/	/
		/	ISKpn24	/	/	/
		/	ISKpn28	/	/	/
		/	ISEhe3	/	/	/
		/	ISKpn2	/	/	/
		/	ISKpn1	/	/	/
		/	ISKpn42	/	/	/
K-DXH20e232	IncF [F-:A-:B-]	IncHI1B	IS5075	/	*tet*(A)	/
		IncFIB(Mar)	/	/	/	/
		/	IS903	/	/	*fimH*, *mrkA*
		/	ISKpn21	/	/	*terC*
		/	ISKpn26	/	/	/
		/	IS5	/	/	/
K-DXH20e233	IncF [F-:A-:B-]	IncA/C2	ISCfr1, ISEc9, ISKpn26	/	*bla*$_{TEM-1B}$, *sul2*, *aac(3)-IId*, *aph(6)-Id*, *aph(3'')-Ib*, *qnrS1*, *bla*$_{CTX-M-15}$	/
		IncFIB(K)	ISKox1, ISKpn42	/	/	/
		Col440I	/	/	/	/
		IncFIB(Mar)	/	/	/	/
		IncHI1B	/	/	/	/

		IncL/M(pOXA-48)	/	/	/	/
		/	ISEcl10	/	*OqxB*, *OqxA*	*fimH*, *mrkA*
		/	ISAeme3, ISAeme4	/	*bla*$_{\text{MOX-6}}$	/
		/	ISKpn38, IS102	/	/	/
		/	ISSen3	/	/	/
		/	IS5075	/	/	/
K-JX19e03	IncF [K2:A-:B-]	IncQ1	/	/	*aph(6)-Id*, *aph(3'')-Ib*, *sul2*	/
		IncFIB(pKPHS1)	/	/	/	/
		IncFII(K)	/	/	/	/
		IncFIB(K)	ISSen4	/	/	/
		/	ISEhe3	/	*OqxB*, *OqxA*	/
		/	ISSty2	/	*bla*$_{\text{SHV-27}}$	/
		/	ISEcl1, IS102	/	/	*clpK1*
		/	IS903	/	/	*iutA*
		/	ISEc15	/	/	/
		/	ISKpn28	/	/	/
		/	ISKpn47	/	/	/
		/	ISKpn1	/	/	/
		/	IS5075	/	/	/
		/	ISEc33	/	/	/
		/	IS26	/	/	/
K-MJD19e39	IncF [F-:A-:B-]	IncN	/	/	/	/
		IncFIB(K)	/	/	/	/
		Col440I	/	/	/	/

		/	ISKpn19	/	*bla*$_{\text{TEM-1B}}$, *tet*(A), *bla*$_{\text{LAP-2}}$, *qnrS1*	/
		/	IS6100	/	*sul1, aac(6')-Ib3, catB3, bla*$_{\text{OXA-1}}$*, aadA16, qacE, aac(6')-Ib-cr*	/
		/	ISAba1	/	*sul2*	/
		/	ISKpn38	/	/	/
		/	ISEcl1	/	/	/
		/	IS1006	/	/	/
		/	ISEcl1	/	/	/
		/	ISKpn8	/	/	/
K-MJD19e29	IncF [K2:A-:B-]	IncFIB(K)	ISSen4	/	/	/
		IncQ1	/	/	*aph(6)-Id*, *aph(3'')-Ib*, *sul2*	/
		IncFIB(pKPHS1)	/	/	/	/
		IncFII(K)	/	/	/	/
		/	ISEhe3	/	*OqxB*, *OqxA*	/
		/	ISSty2	/	*bla*$_{\text{SHV-27}}$	/
		/	IS903	/	/	*iutA*
		/	ISEcl1, IS102	/	/	*clpK1*
		/	ISEc15	/	/	/
		/	ISKpn28	/	/	/
		/	ISKpn1	/	/	/
		/	IS5075	/	/	/
		/	ISKpn47	/	/	/

		/	ISEc33	/	/	/
		/	IS26	/	/	/
K-MJD19e25	IncF [F-:A-:B-]	IncFIB(K)	/	/	/	/
		/	IS1006	/	*qacL*, *mef*(B), *aadA1*, *sul3*, *aadA2*, *cmlA1*, *dfrA12*	/
		/	ISKpn19	/	*bla*$_{\text{TEM-1B}}$, *qnrS1*, *bla*$_{\text{LAP-2}}$, *tet*(A)	/
		/	ISAba1	/	*sul2*	/
		/	ISKpn38	/	/	/
		/	ISEcl1	/	/	/
		/	ISKpn8	/	/	/
K-MJD19e35	IncF [K2:A-:B-]	IncFIB(K)	ISSen4	/	/	/
		IncQ1	/	/	*aph(6)-Id*, *aph(3'')-Ib*, *sul2*	/
		IncFIB(pKPHS1)	/	/	/	/
		Col440I	/	/	/	/
		IncFII(K)	/	/	/	/
		/	ISEhe3	/	*OqxB*, *OqxA*	/
		/	ISSty2	/	*bla*$_{\text{SHV-27}}$	/
		/	ISEcl1, IS102	/	/	*clpK1*
		/	IS903	/	/	/
		/	ISEc15	/	/	/
		/	ISKpn28	/	/	/
		/	ISKpn1	/	/	/

		/	IS5075	/	/	/
		/	ISKpn47	/	/	/
		/	ISEc33	/	/	/
		/	IS26	/	/	/
K-MJD19e32	IncF [F-:A-:B-]	IncFIB(K)	/	/	/	/
		Col440I	/	/	/	/
		/	IS1006	/	*qacL*, *mef*(B), *aadA1*, *sul3*, *aadA2*, *cmlA1*, *dfrA12*	/
		/	ISKpn19	/	*bla*$_{\text{TEM-1B}}$, *qnrS1*, *bla*$_{\text{LAP-2}}$, *tet*(A)	/
		/	ISAba1	/	*sul2*	/
		/	ISKpn38	/	/	/
		/	ISEcl1	/	/	/
		/	ISKpn8	/	/	/
K-BYS20e89	IncF [F-:A-:B-]	IncFIB(K)	ISSen4	/	/	/
		IncFIB(Mar)	ISKpn8, ISKpn41	/	/	/
		Col(MG828)	/	/	/	/
		IncR	/	/	/	/
		/	/	Tn6082	*aph(6)-Id*, *aph(3'')-Ib*	/
		/	ISEhe3	/	*OqxB*, *OqxA*	/
		/	IS26	/	/	*clpK1*
		/	ISKpn24	/	/	/
		/	IS102	/	/	/

		/	ISEcl1,	/	/	/
		/	ISKpn21	/	/	/
		/	ISKpn38	/	/	/
		/	IS5075	/	/	/
		/	ISKpn34	/	/	/
		/	ISKpn26	/	/	/
K-MJD20e148	IncF [K9:A-:B-]	IncFIB(K)	IS903	/	/	/
		IncFII	/	/	/	/
		IncN	/	/	/	/
		Col440I	/	/	/	/
		/	ISVsa3	/	*floR*	/
		/	IS102	/	/	*clpK1*
		/	ISSty2	/	/	/
		/	IS5	/	/	/
		/	ISEcl1	/	/	/
		/	ISEc15	/	/	/
		/	ISKpn28	/	/	/
		/	IS6100	/	/	/
K-MJD20e149	IncF [K9:A-:B-]	IncFIB(K)	IS903	/	/	/
		IncQ1	/	/	*sul2*, *aph(6)-Id*, *aph(3")-Ib*	/
		IncFII(K)	/	/	/	/
		IncN	/	/	/	/
		Col440I	/	/	/	/
		IncFII	/	/	/	/
		Col156	/	/	/	/

		/	ISKpn19	/	*aac(3)-IId*	/
		/	ISVsa3	/	*floR*	/
		/	IS102	/	/	*clpK1*
		/	ISSty2	/	/	/
		/	IS5	/	/	/
		/	ISKpn8	/	/	/
		/	ISKpn47	/	/	/
		/	ISEcl1	/	/	/
		/	ISEc15	/	/	/
		/	ISKpn28	/	/	/
		/	IS6100	/	/	/
K-DXH20e234	IncF [F-:A-:B-]	IncFIB(K)	ISKox1, ISKpn42	/	/	/
		IncHI1B	/	/	/	/
		/	ISKpn21	/	/	/
		/	ISKpn24, ISSen3	/	/	/
		/	IS5075	/	/	/
		/	ISKpn38	/	/	/
		/	ISKpn43	/	/	/
		/	/	Tn6082	/	/
		/	ISEhe3	/	/	/
		/	IS102	/	/	/
KP-HB21H30786	IncF [F33:A-:B-]	ColRNAI	/	/	*ccI*	/
		IncFII(pHN7A8)	/	/	/	/
		IncR	/	/	/	/
		/	ISEc9	/	*bla*$_{SHV-182}$	/
		/	ISKpn18	/	*fosA*	/

		/	ISKpn27	/	*bla*$_{KPC-2}$	/
		/	ISVsa3	/	*tet*(X4)	/
		/	ISCfr1	/	/	/
		/	ISKpn1	/	/	/
		/	IS5075	/	/	/
		/	ISKpn28	/	/	/
		/	IS26	/	/	/
		/	ISKpn14	/	/	/
KP-HB21H30727	IncF [K34:A-:B-]	ColRNAI	/	/	/	*ccI*
		IncFIB(K)	/	/	/	/
		/	ISEc9	/	*bla*$_{TEM-1B}$, *bla*$_{CTX-M-15}$	/
		/	ISKpn27	/	*bla*$_{KPC-2}$	/
		/	ISEch12	/	/	/
		/	ISKpn33	/	/	/
		/	IS5075	/	/	/
		/	ISVsa3	/	/	/
		/	ISEcl1	/	/	/
		/	ISKpn26	/	/	/
		/	ISKpn14	/	/	/
KP-HB21H30799	IncF [F-:A-:B-]	IncFIB(pKPHS1)	/	/	/	/
		IncR	/	/	/	/
		/	ICEEcoED1a-1, ISKpn1, IS102	/	/	*irp2*, *fyuA*
		/	IS5075	/	/	/
		/	ISKpn26	/	/	/

		/	IS26	/	/	/
KP-HB21H30845	IncF [F33:A-:B-]	IncHI1B	IS903	/	/	*iucC*, *iutA*
		IncFIB(K)	ISKox1, ISKpn42	/	/	/
		ColRNAI	/	/	/	*ccI*
		IncFIB(pKPHS1)	/	/	/	/
		IncFII(pHN7A8)	/	/	/	/
		lncR	/	/	/	/
		/	ISKpn19	/	*qnrS1*, *bla*$_{\text{LAP-2}}$	/
		/	ISKpn27	/	*bla*$_{\text{KPC-2}}$	/
		/	ISVsa3	/	*sul2*	/
		/	ISCfr1	/	/	/
		/	ISKpn1	/	/	/
		/	IS5075	/	/	/
		/	ISKpn18	/	/	/
		/	ISKpn28	/	/	/
		/	ISKpn14	/	/	/
KP-HB21H30826	IncF [F-:A-:B-]	lncHI1B	ISEhe3, ISKpn2, IS102	/	/	*iroN*
		IncFIB(K)	/	/	/	/
		/	ISCfr13	/	/	/
		/	ISKpn1	/	/	/
KP-HB21H30761	IncF [F-:A-:B-]	IncFIB(K), IncHI1B	ISKpn2			
		/	ISSty2	/	*bla*$_{\text{SHV-199}}$, *bla*$_{\text{SHV-26}}$, *bla*$_{\text{SHV-179}}$,	/

					bla$_{SHV-194}$, *bla*$_{SHV-78}$, *bla*$_{SHV-98}$, *bla*$_{SHV-145}$	
		/	ISKpn38	/	/	*irp2*, *fyuA*
		/	ISSen4	/	/	/
		/	ISEhe3	/	/	/
		/	ISCfr13	/	/	/
		/	IS102	/	/	/
KP-HB21H30535	IncF [F33:A-:B-]	IncHI1B	IS903	/	/	*iutA*, *terC*, *iucC*
		IncFIB(K)	ISKox1, ISKpn42	/	/	/
		ColRNAI	/	/	/	*ccI*
		IncFIB(pKPHS1)	/	/	/	/
		IncR	/	/	/	/
		IncFII(pHN7A8)	/	/	/	/
		/	ISVsa3	/	*sul2*	/
		/	ISKpn19	/	*bl*$_{aLAP-2}$, *qnrS1*	/
		/	ISKpn27	/	*bla*$_{KPC-2}$	/
		/	IS102	/	*bla*$_{CTX-M-65}$	/
		/	ISCfr1	/	/	/
		/	ISKpn1	/	/	/
		/	IS5075	/	/	/
		/	ISKpn18	/	/	/
		/	ISKpn28	/	/	/
		/	ISKpn14	/	/	/
KP-HB21H30813	IncF [K2:A-:B-]	lncHI1B	ISKpn2	/	/	*terC*

		IncFIB(K)	/	/	/	/
		IncFII(K)	/	/	/	/
		/	IS6100	/	*mph*(A), *qacE*, *dfrA27*, *qnrB91*, *sul1*, *ARR-3*, *aadA16*, *sul1*, *aac(6')-Ib-cr*	/
		/	ISKpn47	/	/	*traT*
		/	ISKpn38	/	/	*fyuA*, *irp2*
		/	ISEhe3	/	/	/
		/	ISKpn1	/	/	/
		/	ISSen4	/	/	/
		/	IS26	/	/	/
KP-HB21H30772	IncF [F-:A-:B-]	IncFIB(K), IncHI1B	ISKox1, ISKpn42, IS903	/	/	*terC*,*iutA*, *iucC*
		/	ISSty2	/	/	/
		/	ISKpn1	/	/	/
		/	ISKpn28	/	/	/
		/	IS102	/	/	/
KP-HB21H30740	IncF [K5:A-:B-]	IncFIB(K), IncFII(K)	ISVsa3, ISKpn14, ISKpn19, IS903, IS26, ISKpn14	/	*aph(6)-Id*, *tet*(A), *floR*, *aph(3'')-Ib*, *sul2*	*traT*, *iucC*, *iutA*
		Col440I	/	/	/	/
		/	ICEEcoED1a-1	/	/	*fyuA*, *irp2*
		/	ISEch12	/	/	/
		/	IS5075	/	/	/

K-210828y126.V-2	IncF [K5:A-:B-]	IncFII(K), IncFIB(K)	ISKpn14, ISKpn19, ISEc45, IS903, IS26	/	*qnrS1*, *bla*$_{\text{LAP-2}}$	*iucC*, *iutA*, *traT*
		Col440II	/	/	/	/
		/	ISEch12	/	/	/
K-20210828y126.M-2	IncF [K5:A-:B-]	IncFII(K)	ISKpn19, IS903, IS26	/	*qnrS1*, *bla*$_{\text{LAP-2}}$	/
		IncFIB(K)	ISEc45, ISKpn14	/	/	*iucC*,*traT*, *iutA*
		Col440I	/	/	/	/
		/	ISEch12	/	/	
K-20210828y196.M-1	IncF [K5:A-:B-]	IncFII(K), IncFIB(K)	ISKpn14, ISKpn19, ISEc45, IS903, IS26 ISEch12		*qnrS1*, *bla*$_{\text{LAP-2}}$	*iucC*, *traT*, *iutA*
K-20210828y196.M-4	IncF [K5:A-:B-]	IncFIB(K), IncFII(K)	ISKpn14, ISKpn19, ISEc45, IS903, IS26	/	*qnrS1*, *bla*$_{\text{LAP-2}}$	*iucC*, *traT*, *iutA*
		Col156	/	/	/	/
		/	ISEch12	/	/	/
K-210830y115.M-5	IncF [K5:A-:B-]	IncFII(K)	ISKpn19, IS903, IS26	/	*qnrS1*, *bla*$_{\text{LAP-2}}$	/
		IncFIB(K)	ISEc45, ISKpn14	/	/	*iutA*, *traT*, *iucC*
		/	ISEch12	/	/	/
K-210902y113.M-5	IncF [K5:A-:B-]	IncFIB(K)	ISEc45, IS903	/	/	*iutA*, *iucC*
		/	ISVsa3	/	*sul2*, *aph(6)-Id*, *tet*(A), *floR*,*aph(3'')-Ib*	/
			ISKpn19	/	*bla*$_{\text{LAP-2}}$, *qnrS1*	/
			ISKpn14	/	/	*traT*

			ICEEcoED1a-1	/	/	*fyuA*, *irp2*
			ISEch12	/	/	/
			IS5075	/	/	/
			IS26	/	/	/
K-210908y245-v-m1	IncF [K5:A-:B-]	IncFIB(K), IncFII(K)	ISKpn14, ISEc45	/	/	*iucC*, *traT*, *iutA*
		IncX1	/	/	/	/
		Col156	/	/	/	/
K-210908y408-2v-m1	IncF [K5:A-:B-]	IncFIB(K)	ISKpn14, ISEc45, ISKpn14	/	/	*iucC*, *traT*, *iutA*
		IncX1	/	/	/	/
		Col156	/	/	/	/
K-211002y45204V-2-M	IncF [K3:A-:B-]	/	ISSty2		$bla_{SHV-199}$, bla_{SHV-78}, bla_{SHV-98}, bla_{SHV-26}, $bla_{SHV-179}$, $bla_{SHV-145}$, $bla_{SHV-194}$	/
		IncFII(K)	IS640	/	*iutA*, *iucC*	/
		IncFIB(AP001918)	ISKpn28	/	/	/
		IncFIA(HI1)	/	/	/	/
		/	ISSen4, ISSty2	/	/	/
		/	IS26	/	/	/
K-211002y64V-1-M	IncF [F-:A-:B-]	IncFIB(K)	ISKox1, ISKpn42	/	/	/
		/	IS1006	/	*dfrA12*, *qacE*,	/

					floR, *sul1*, *aadA2*	
			ISEhe3	/	/	*nlpI*
			ISKpn38, IS102	/	/	/
			ISKpn24	/	/	/
			ISSm1	/	/	/
			IS26	/	/	/
KQ-211002y64V-2-M	IncF [F-:A-:B-]	IncFIB(K)	/	/	/	/
		IncFII(K)	/	/	/	
		/	IS1133	/	*aph(6)-Id*, *aph(3'')-Ib*	/
			ISVsa3	/	*floR*, *tet*(A)	/
			IS903	/	/	/
			ISKpn34	/	/	/
			ISKox1	/	/	/
			IS26	/	/	/
KP00519	IncF [F-:A-:B-]	IncFIB(K), IncFII(K)	ISKpn14, ISKpn19, ISEc45, IS903, IS26	/	*qnrS1*, *bla*$_{\text{LAP-2}}$	*traT*, *iucC*, *iutA*
		/	ISEch12	/	/	/
KP00520	IncF [F-:A-:B-]	IncFIB(K), IncHI1B	ISKox1, ISKpn42, IS903	/	/	*terC*
		Col440I	/	/	/	/
		/	ISEcl10	/	*bla*$_{\text{SHV-26}}$	/
		/	ISSty2	/	*OqxB*, *OqxA*	/
		/	ISEhe3	/	/	/
		/	IS102	/	/	/
KP00521	IncF [F-:A-:B-]	IncR	IS903	/	/	/

		IncN	/	/	/	/
		Col440I	/	/	/	/
		/	IS6100	/	*ARR-3*, *mph*(A), *qacE*, *sul1*, *dfrA27*, *qacE*, *qnrB6*, *aadA16*, *sul1*, *aac(6')-Ib-cr*	/
		/	ISEhe3	/	*OqxB*, *OqxA*	/
		/	ISVsa3	/	*floR*	/
		/	ISKpn1	/	/	/
		/	/	Tn801	*bla*$_{\text{TEM-1A}}$, *sul2*, *aph(6)-Id*, *aph(3'')-Ib*	/
KP00522	IncF [K5:A-:B-]	IncFII(K), IncFIB(K)	ISKpn14, ISKpn19, ISEc45, IS903, IS26	/	*qnrS1*, *bla*$_{\text{LAP-2}}$	*iucC*, *iutA*, *traT*
		/	ISEch12	/	/	/
KP00523	IncF [F-:A-:B-]	IncFIB(K), IncHI1B	ISKox1, ISKpn42, IS903	/	/	*terC*
		Col440I	/	/	/	/
		/	ISEcl10	/	*bla*$_{\text{SHV-26}}$	/
		/	ISSty2	/	*OqxB*, *OqxA*	/
		/	ISEhe3	/	/	/
		/	IS102	/	/	/
KP00524	IncF [F-:A-:B-]	IncR	ISKpn19		*qacE*, *qnrS1*, *bla*$_{\text{LAP-2}}$, *tet*(A),	/

					sul1, *dfrA1*	
		ColRNAI	/	/	/	/
		IncFIB(pKPHS1)	/	/	/	/
		Col440II	/	/	/	/
		rep22	/	/	/	/
		/	ISEch12	/	/	/
		/	ISSen4	/	/	/
		/	ISKpn43	/	/	/
KP00525	IncF [F-:A-:B-]	IncN	/	/	/	/
		IncR	/	/	/	/
		Col440I	/	/	/	/
		/	IS6100	/	*qacE*, *mph*(A), *dfrA27*, *qnrB6*, *aadA16*, *ARR-3*, *aac(6')-Ib-cr*, *sul1*	/
		/	/	Tn801	*sul2*, *aph(6)-Id*, *aph(3'')-Ib*	
		/	ISEhe3	/	*OqxB*, *OqxA*	*fimH*, *mrkA*
		/	ISVsa3	/	*floR*	/
		/	IS903	/	/	/
		/	ISKpn1	/	/	/
		/	IS26	/	/	/

Table S5. Virulence-related genes of *52 K. pneumoniae* strains.

**Strains**	**Yersiniabactin**	**Aerobactin**	**Salmochelin**	**Hypermucoidy genes**
K-BYS20c105	/	/	/	/
K-BYS20c109	/	/	/	/
K-MJD20e150	/	/	/	/
K-MJD20c164	*ybt5* (ICEKp6)	/	/	/
K-MJD20b201	*ybt7* (ICEKp7)	/	/	/
K-DXH20e220	/	/	/	/
K-DXH20e225	/	/	/	/
K-DXH20e226	/	/	/	/
K-DXH20e227	/	/	/	/
K-DXH20e228	/	/	/	/
K-DXH20e230	/	/	/	/
K-DXH20e231	/	/	/	/
K-DXH20e232	/	/	/	/
K-DXH20e233	/	/	/	/
K-JX19e03	/	/	/	/
K-MJD19e39	/	/	/	/
K-MJD19e29	/	/	/	/
K-MJD19e25	/	/	/	/
K-MJD19e35	/	/	/	/
K-MJD19e32	/	/	/	/
K-BYS20e89	/	/	/	/
K-MJD20e148	/	/	/	/
K-MJD20e149	/	/	/	/
K-DXH20e234	/	/	/	/
KP-HB21H30786	*ybt9* (ICEKp3)	/	/	/
KP-HB21H30727	*ybt14* (ICEKp12)	/	/	/
KP-HB21H30799	*ybt1* (ICEKp4)	/	/	/
KP-HB21H30845	*ybt9* (ICEKp3)	*iuc1*	/	*rmpA*
KP-HB21H30826	*ybt4* (plasmid)	/	*iro1*	/
KP-HB21H30761	*ybt4* (plasmid)	/	*iro1*	/
KP-HB21H30535	*ybt9* (ICEKp3)	/	*iro1*	/
KP-HB21H30813	*ybt4* (plasmid)	/	/	/
KP-HB21H30772	*ybt22* (ICEKp12)	*iuc1*	*iro1*	*rmpA*
KP-HB21H30740	*ybt1* (ICEKp4)	*iuc3*	/	/
K-210828y126.V-2	*ybt15* (ICEKp11)	*iuc3*	/	/
K-20210828y126.M-2	*ybt15* (ICEKp11)	*iuc3*	/	/
K-20210828y196.M-1	*ybt15* (ICEKp11)	*iuc3*	/	/
K-20210828y196.M-4	*ybt15* (ICEKp11)	*iuc3*	/	/
K-210830y115.M-5	*ybt15* (ICEKp11)	*iuc3*	/	/
K-210902y113.M-5	*ybt1* (ICEKp4)	*iuc3*	/	/
K-210908y245-v-m1	/	*iuc3*	/	/

K-210908y408-2v-m1	/	*iuc3*	/	/
K-211002y45204V-2-M	/	*iuc* (unknown)	/	/
K-211002y64V-1-M	/	/	/	/
KQ-211002y64V-2-M	/	/	/	/
KP00519	*ybt15* (ICEKp11)	*iuc3*	/	/
KP00520	/	*iuc1*	/	/
KP00521	/	/	/	/
KP00522	*ybt15* (ICEKp11)	*iuc3*	/	/
KP00523	/	*iuc1*	*iro1*	/
KP00524	/	/	/	/
KP00525	/	/	/	/

Table S6. Prevalence distribution of *K. pneumoniae* in farm animals.

Type of farm	Region	Farm numbers	No. samples	No. isolates	Proportion (%)
Bovine	Hubei	4	126	24	19.0
Porcine	Hunan	1	85	11	12.9
Duck	Hubei	6	59	7	11.9
